# New ultra-sensitive radioanalytical technologies for new science

**DOI:** 10.1007/s10967-018-5787-3

**Published:** 2018-03-14

**Authors:** Pavel P. Povinec

**Affiliations:** Department of Nuclear Physics and Biophysics, Faculty of Mathematics, Physics and Informatics, Comenius University, Mlynska dolina F1, 84248 Bratislava, Slovakia

**Keywords:** Radionuclides, Ge detector, Underground laboratory, Accelerator mass spectrometry, Atmosphere, Marine environment

## Abstract

Recent developments in radiometric and mass spectrometry technologies have been associated in the radiometric sector mainly with underground operations of large volume Ge detectors, while the mass-spectrometry sector, represented mainly by accelerator mass spectrometry and inductively coupled plasma mass spectrometry has become the most sensitive technique for ultra-low-level analyses of long-lived radionuclides. These new developments have had great impact on investigations of rare nuclear processes and applications of radionuclides in environmental, life and space sciences. New scientific investigations have been carried out therefore which have not been possible before either because of lack of sensitivity or required large sample size.

## Introduction

Radioanalytical technologies have always been a limiting factor for experiments in nuclear sciences comprising investigations of rare processes in nuclear physics and chemistry, in space research, in environmental radioactivity studies, in isotope oceanography and hydrology, in biomedical research and in many other branches of science. This has been mainly because of the fact that the available sensitivity was not high enough to get meaningful results, or the required sample size was too big to carry out such investigations [1–4]. Environmental radionuclide tracer studies began about 60 years ago with applications of cosmogenic (^14^C and ^3^H) and radiogenic (^210^Pb, ^226^Ra) radionuclides. This has been a rapidly developing field, which included studies on behaviour of radionuclides in the environment, use of natural and anthropogenic radionuclides as tracers of environmental processes, marine radioactivity studies, radiation protection, radioecology, etc., to mention at least a few of them. These studies have always been limited by the techniques available for sampling and analysis of radionuclides in the environment. As the levels of radionuclides observed recently in the environment have been very low, high sensitive radioanalytical systems have been required for carrying out new environmental investigations.

Traditionally radionuclide analyses have been carried out using alpha-, beta-, and gamma-spectrometers, jointly called radiometric counting systems. In the field of radiometric analytical technologies we moved from simple radiochemical radionuclide separation methods and gas counters to efficient robotic radiochemical techniques and sophisticated detectors working on line with powerful computers. The radiation detectors have often been situated underground, or they have been using anticosmic/antiCompton shielding to protect them against cosmic radiation. Such arrangements have considerably decreased the detectors background, and thus increased their sensitivity for analysis of radionuclides at very low levels in various types of samples.

Simultaneously, the philosophy of sampling and laboratory measurements has changed, where appropriate, to in situ analysis of radionuclides in the air, on land, in water and in the sediment, thus enabling developments of concentration maps and/or time series on radionuclide distributions in the investigated environments [1, 3, 5–13]. This has been a complimentary detection method to a traditional sampling and laboratory analysis, which can have several advantages, e.g. it can avoid a complicated and laborious sampling, pre-concentration and separation works, and long-term measurements are not necessary, especially when mapping of gamma-emitters in large surface areas would be required. The systems commonly used for underwater gamma-spectrometry are mostly based on NaI(Tl) detectors, with one exception when a methane cooled Ge detector was used [5, 6, 8]. The advantage of NaI(Tl)-based systems is firstly related to the high detection efficiency of NaI(Tl) crystals at much lower cost than equivalent Ge crystals. The drawbacks of NaI(Tl) systems are the high-power consumption for the operation of the photomultiplier tube, and a relatively poor energy resolution. The Ge-based systems have the advantage of good energy resolution, and hence excellent radionuclide identification capability. Other available types of detectors—crystals and semiconductors have not become competitive with NaI(Tl) and Ge detectors mainly because of their lower efficiency for gamma-rays in the energy interval of 30–3000 keV. The in situ technology has enabled to carry out not only radionuclide mapping experiments (for example using a towed detector system placed on a boat [9, 10]), but also to develop on line monitoring systems, either for monitoring of radionuclide releases from nuclear installations, investigations of changes in oceanic current systems [13], as well as for monitoring radon decay products in submarine groundwater discharge studies [14–17].

There have been several motivations for new developments of laboratory-based ultra-sensitive radioanalytical techniques in nuclear and environmental studies: (i) Levels of anthropogenic radionuclides after over 60 years of their injection to the environment have decreased considerably [1–4, 18–20]; (ii) Sample size required for radiometric analyses should be comparable to mass spectrometry analyses (e.g. in seawater profile sampling Rosette systems with 10–20 L bottles should be used instead of large 200 L (for ^137^Cs) or 400 L (for Pu isotopes) sampling containers [1, 3, 4, 21–26]; (iii) Highly accurate, precise and traceable data are required for environmental and climate change studies, which would require detection limits below 1 nBq/g [1–4, 27–31]; (iv) New scientific ideas—such as investigations of rare nuclear processes and decays, investigations of cosmic dust, solar variations, supernova explosions, deep-sea bottom studies, DNA studies, environmental biotechnology, environmental nanotechnology, climate change studies, etc., have not been possible to realize till now as they have been requiring new ultra-sensitive radioanalytical technologies [32–38], with sensitivities good enough to analyse even very small samples.

The recent developments in low-level radioanalytical techniques have helped to improve the detection limits using: (i) High efficiency Ge detectors (up to 200% of relative efficiency compared to 76 mm in diameter and 76 mm long NaI(Tl) crystal) operating in underground laboratories which represent the most important achievement in radiometric analysis of radionuclides [1–4, 18–26, 35, 36]; (ii) Anticosmic shielding of detectors operating in surface and shallow-depth underground laboratories [39–42] which helped to improve detection limits comparable to deeper underground laboratories; (iii) Multidetector coincidence gamma-spectrometry systems for analysis of cascade and/or positron emitters [43–51]; (iv) Multidimensional gamma-spectrometry [52, 53]. Thanks to excellent energy resolution of Ge detectors (< 2 keV for 1.33 MeV gamma-rays), and possibility to analyse various gamma-emitters in material and environmental samples selectively and very often non-destructively, they dominated in the field of ultra-low-level gamma-spectrometry [54–60]. If such spectrometers can operate at least a few tens of meters underground, their performance can be superior in comparison with laboratories operating at the surface [39, 40, 47, 56, 60]. However, the underground gamma-spectrometry techniques are restricted to gamma-emitters only such as ^7^Be, ^40^K, ^54^Mn, ^60^Co, ^137^Cs, ^210^Pb, and many others. For analysis of beta-emitters found in the environment (e.g. ^3^H, ^14^C, ^81^Kr, ^85^Kr, ^90^Sr, ^133^Xe, ^135^Xe, ^241^Pu, and others), gas counting [61–77] and liquid scintillation spectrometers have been used [37, 78–80]. In some applications, however, radiometric techniques of analysis of long-lived beta-emitters did not allow to carry out state of the art nuclear and environmental research either because of lack of sensitivity or requirement of large samples for analysis.

The most important break-through in the radioanalytical technologies has been, however, a general change in philosophy of radionuclide analysis. We moved from the concept of counting of radioactive decays (and thus waiting for them for a long time in the case of long-lived radionuclides) to the direct counting of atoms (as they would be stable) using high-sensitive mass spectrometers working either with low-energy ions (e.g. Inductively Coupled Plasma Mass Spectrometry—ICPMS [81–85], Resonance Ionization Mass Spectrometry—RIMS [86, 87], Thermal Ionization Mass Spectrometry—TIMS [88, 89], Secondary Ionization Mass Spectrometry—SIMS) [2, 90], or with ions accelerated up to hundreds of MeV in accelerator mass spectrometry (AMS) systems [91–98]. These developments have considerably improved the detection limits for analysis of radionuclides in all scientific applications requiring ultra-high radionuclide sensitivities and small sample size.

Two mass-spectrometry systems have recently been dominating in the field of ultra-sensitive radionuclide research, namely AMS and ICPMS, thanks to their ultra-high sensitivity, accuracy and precision, as well as to their ability to reach high output rates in analyses of long-lived radionuclides in all types of samples. The AMS dominated in analyses of several long-lived radionuclides such as ^10^Be, ^14^C, ^26^Al, ^41^Ca, ^129^I, U and Pu isotopes, and others [91–98]. For analysis of some other long-lived radionuclides (e.g. ^99^Tc, ^129^I, U and Pu isotopes), the ICPMS has recently been representing, however, also very important breakthrough.

Another important trend which we should mention for applications of radionuclides in environmental sciences is connected with a transfer from a simple bulk sample analysis to specific compound analysis of stable and radioactive isotopes, very often resulting in use of coupled analytical systems, e.g. coupling of gas chromatography with AMS systems. By a combination of gas chromatographs, which select and separate necessary amounts of specific compounds, with an AMS which perform mass isotope analysis, it has been possible to open new windows for new isotope research [98–101]. This trend is having, however, more general applications, e.g. in geochemistry and cosmochemistry, where move from bulk radionuclide analyses of samples to specific radionuclide analysis of minerals would be feasible. More attention should also be given to analyses of radionuclides on particles, and generally on speciation studies of radionuclides in the environment [3].

Sample sizes have been constraining many new environmental investigations, which could be illustrated e.g. for surface seawater sampling and analysis: (i) 0.5 L sample size limit has already been achieved for ^3^H analysis using in-growth mass spectrometry of ^3^He [2, 102, 103] and in AMS analysis of ^14^C and ^129^I [2, 92, 93, 98, 102–105]; (ii) About 5 L seawater samples could be analysed for ^137^Cs in deep-underground gamma-spectrometry laboratories such as Gran Sasso (Italy) and Modane (France); (iii) About 10 L samples could be analysed for ^137^Cs in shallow and medium-depth underground gamma-spectrometry laboratories, operating at about 100 m water equivalent, w.e. (for comparison of laboratories operating in different rock environment we normalize them to the water depth) [60, 106–108]; (iv) About 10 L samples could be analysed for Pu isotopes by AMS [60, 96, 109, 110], and similarly also by ICPMS and TIMS [81, 88, 97, 110]. These developments in the radioanalytical technologies have had important impacts on sampling strategies as well. Due to over two orders of magnitude decrease in the sampling size it has been possible to sample e.g. even deep-sea water columns using conventional Rosette systems during one/two casts only. This enabled to carry out high resolution radionuclide water profile studies, which were not realizable before using conventional large volume water samplers (200–500 L), just because of sampling and financial constraints [1–4, 60, 106–109]. This move from simple radioanalytical techniques to the present sophisticated state of the art technologies has also been accompanied by a considerable change in the philosophy of environmental studies as well. We moved from institutional environmental investigations to global international projects carried out during recent years, e.g. WOCE (World Ocean Circulation Experiment), CLIVAR (Climate Variability and Predictability), PAGES (Past Global Changes), WOMARS (World Ocean Marine Radioactivity Studies), SHOTS (South Hemisphere Ocean Tracer Studies), GEOTRACES (Geochemical Traces in the oceans) to mention at least a few of them.

We shall discuss in this review developments in ultra-sensitive radionuclide analysing techniques, focusing on radiometric and mass spectrometry methods. We shall shortly follow developments in radiocarbon measuring techniques from the Libby counter through proportional gas counters and liquid scintillation spectrometers to more recent developments of AMS. While during the first 60 years of the radiocarbon measurements the beta-counting, specifically the gas counting was the dominant technique, in the present the dominant technology in radiocarbon science is AMS. We shall also focus on gamma-spectrometry, on the development of large volume Ge detectors very often operating in shallow and deep underground laboratories. Monte Carlo simulations of detector background characteristics have been important pre-requisite when designing low-level counting systems, operating hundreds of meters underground, where radioactive purity of construction materials and radon concentration in the air has become dominant factors controlling the detector background. Mass spectrometry technologies, mainly developments and applications of AMS and ICPMS for environmental studies and for radiopurity measurements of construction parts of large-scale underground nuclear physics experiments will also be discussed in detail. The radiometric and mass spectrometry technologies will be compared with each other and with neutron activation (NAA) methods, which, especially in radiopurity measurements, represent a renaissance of this method for ultra-low-level uranium and thorium analysis in construction materials. These new developments in mass and radiometric spectrometry for ultra-low-level radionuclide analyses have had great impact on investigations of rare nuclear processes and applications in environmental, life and space sciences. We shall present at least a few examples (SuperNEMO experiment, isotope groundwater hydrology and radionuclide tracing in the marine environment). As the topic is very wide, it has not been possible to cover all aspects of sampling and radionuclide analyses in detail; the emphasis has been on recent developments in the field. More information can be found in already published review papers [1–4, 60, 112, 114, 115]. Similarly, the list of references, although very comprehensive, could not cover all the work done in this field, but it is mostly listing our recent publications, including book and book chapters [1–4, 112–115]. A few monographs and book chapters published by other authors have also been included to help readers to find more specific information [91, 94–96, 116–124].

I also organised several conferences on low-level counting and spectrometry with published proceedings, where more detailed information can be found [125–131]. Later, the low-level conferences have been regularly organized by the ICRM (proceedings published in Applied Radiation and Isotopes). For the environmental aspects, I co-organised conferences on environmental radioactivity ENVIRA (proceedings published in Journal of Environmental Radioactivity and in proceedings books). Many conferences were organized on radioanalytical/radiochemistry aspects (e.g. MARC-Kona, RADCHEM-Marianske Lazne, RANC-Budapest, with proceedings published in Journal of Radioanalytical and Nuclear Chemistry). AMS conferences had proceedings regularly published in Nuclear Instruments and Methods Sect. B).

We hope by publishing this paper to offer readers an overview of recent developments in ultra-sensitive radioanalytical technologies and their applications in nuclear and environmental sciences, to encourage and advice-them how to build the state of the art radioanalytical laboratories for nuclear and environmental research. As the paper contains many abbreviations and technical terms, Table 1 lists at least some of them which have been used frequently.

**Table 1:** Abbreviations and technical terms used in the paper

Abbreviation/term	Explanation
TU	Tritium Unit: 1 TU = 10^−18^ ^3^H/^1^H, Eq. 0.118 Bq L^−1^ of water
pMC	Percent Modern Carbon is used for recent environmental samples, calculated against a reference sample of ^14^C activity from a known standard; 100 pMC is ^14^C activity for radiocarbon age of 0 y BP (before present, i.e. before 1950)
∆^14^C	∆^14^C = δ^14^C − 2(δ^13^C + 25)(1 + δ^14^C/1000) (‰) δ^14^C = [(^14^C_sample_ − ^14^C_standard_)/^14^C_standard_] × 10^3^ (‰)
AMS	Accelerator mass spectrometry
ICPMS	Inductively-coupled plasma mass spectrometry
TIMS	Thermal ionization mass spectrometry
PIXE	Particle-induced X-ray emission
IAEA	International Atomic Energy Agency

## Development of radiometric technologies

### Gas counting systems

My scientific carrier started with development of methods for analysis of radiocarbon in atmospheric carbon dioxide [65], and at the beginning it was heavily influenced by W. Libby, D. Lal, K. Münnich, H. Oeschger and many other radiocarbon scientists. There have been several important breakthroughs in the radiocarbon technologies, starting from the Libby counter in which the cathode was covered with carbon sample [132], followed by proportional gas counters [133–135], liquid scintillation spectrometers [136, 137], and finally developing AMS [138–140], which shifted the analytical concept from counting of ^14^C decay products (and waiting for beta-electrons) into direct counting of ^14^C atoms present in a sample. As the half-life of ^14^C is relatively long (5730 year), the number of ^14^C atoms present in a sample compared to number of ^14^C beta-decays observed during one day of counting is ~ 3 × 10^6^, significantly in favour of AMS. Recently we moved into a stage when a bulk sample analysis has been replaced by compound specific analysis, e.g. in gas chromatography-AMS coupled analytical systems, which opened new dimensions in the radiocarbon science [98].

A traditional method of activity measurement using a low current ionization chamber would not give meaningful results for radiocarbon dating because of high background. It was fortunate that gas counters for direct counting of pulses originating in the radiation detector as a result of radioactive decay of nuclei and emission of beta-particles were already in use. Libby [132] introduced a detector shielding to decrease its background (20 cm of iron), but this was not enough because a further, at least by a factor of ten decrease was required to get a reasonable background. Libby recognized that the hard component of cosmic rays (muons), penetrating even the heavy iron shield should be at least partially eliminated. Such a trigger was used for the first time by Blackett and Occhialini [141] to identify tracks of positrons in a cloud chamber. Libby found a revolutionary solution in using a similar trigger, however, in an anticoincidence regime, which eliminated pulses coming from the central detector, if they were simultaneously registered by Geiger tubes surrounding the central detector (i.e. the anticosmic veto). This arrangement decreased the counter background by a factor of twenty, and was good enough to proceed with regular radiocarbon measurements. It has been clear from the beginning of radiocarbon measurements that for achieving larger radiocarbon data outputs, and to ensure better precision, the Libby counter should be replaced by another type of a detector. Because of low energy of ^14^C beta-electrons (their maximum energy is only 156 keV), the best solution would be to incorporate a sample in a form of a gas directly used in the detector. Therefore, it was natural to use carbon dioxide, the first product of sample combustion, which contains ^14^C atoms from the sample. After several trials, this has been achieved by de Vries and Barendsen [133], although it has been recognized that carbon dioxide must be well cleaned to get reasonable counting characteristics. Simultaneously Suess [134] developed a method for preparation of acetylene, and Burke and Meinschein [135] for preparation of methane as suitable ^14^C counting gases. Later we found that the cleaning of CO_2_ from impurities such as water vapours, nitrogen and sulphur oxides, and halogen compounds is required due to its high sensitivity to electronegative impurities, which decreased the transit time of electrons from a place of their origin to the anode by about a factor of ten when compared with CH_4_ [71]. As we predicted that the best counting characteristics should have CH_4_, we constructed an apparatus for preparation of  CH_4_ [142], following the Lal’s design [143]. Further advantages of using CH_4_ were that we could use the same method for ^3^H as well as for ^14^C measurements, which was naturally later enlarged to simultaneous ^3^H and ^14^C counting in the same CH_4_ gas filling [70, 74]. This method has been very promising, and even nowadays it has been used for simultaneous ^3^H and ^14^C measurements in natural methane [144].

**Fig. 1:**
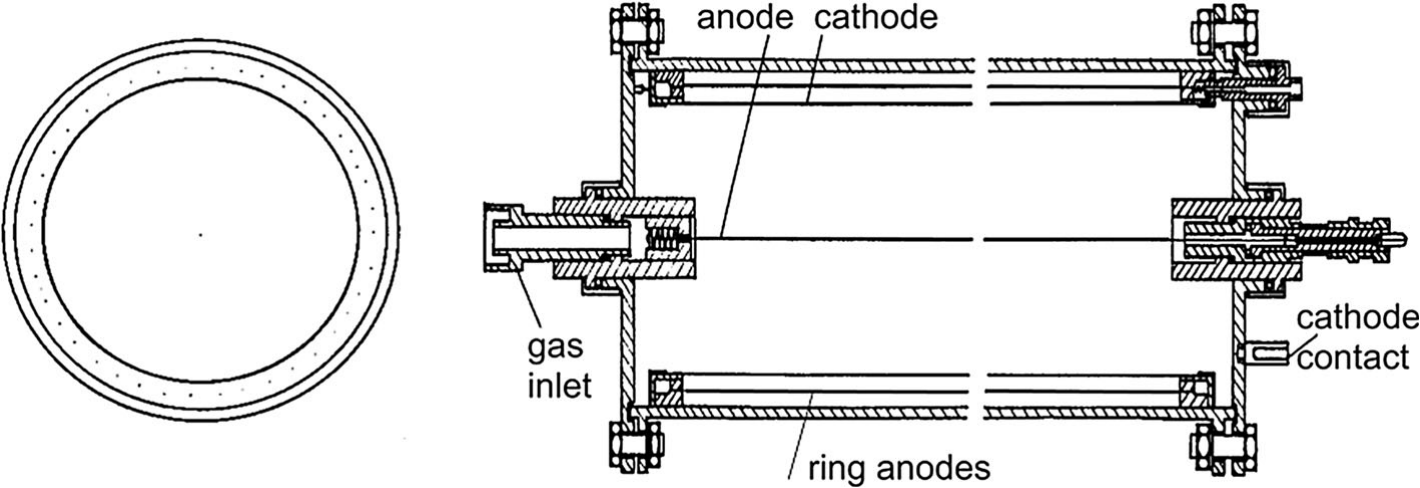
Low-background proportional counter with thin copper cathode for ^3^H and ^14^C counting [68]

It has also been well known from building first ^14^C counters that only radioactivity free construction materials should be used for construction of low-background gas counters (e.g. quartz, electrolytic copper). After a careful analysis of background components of proportional counters, it has been found that the most important contribution to the background of the central detector is the thickness and the material of the inner cathode. The inner cathode should be very thin so the contribution of δ-electrons originating in the cathode by interactions of gamma-rays with the cathode material should be minimized [145]. This became feasible with introduction of a multiwire guard counter, surrounding the inner detector, so only a thin cathode foil could separate both detectors [68, 145] (Fig. 1). A detector with a lowest background should have an optimized thickness of the inner cathode, just for stopping beta-electrons originating from radioactive decay of e.g. ^14^C (in the case of ^14^C beta-electrons the optimum thickness is ~ 10 mg/cm^2^). The cathode should be made from radioactively pure foil (e.g. gold coated Mylar foil or thin copper). In the case of ^3^H counting (because of small energy of beta-electrons, max. 18.6 keV), the internal cathode foil may be even replaced by wires [68]. If the sensitivity of the detector should be increased, the detector could operate under higher than atmospheric pressure.

**Fig. 2:**
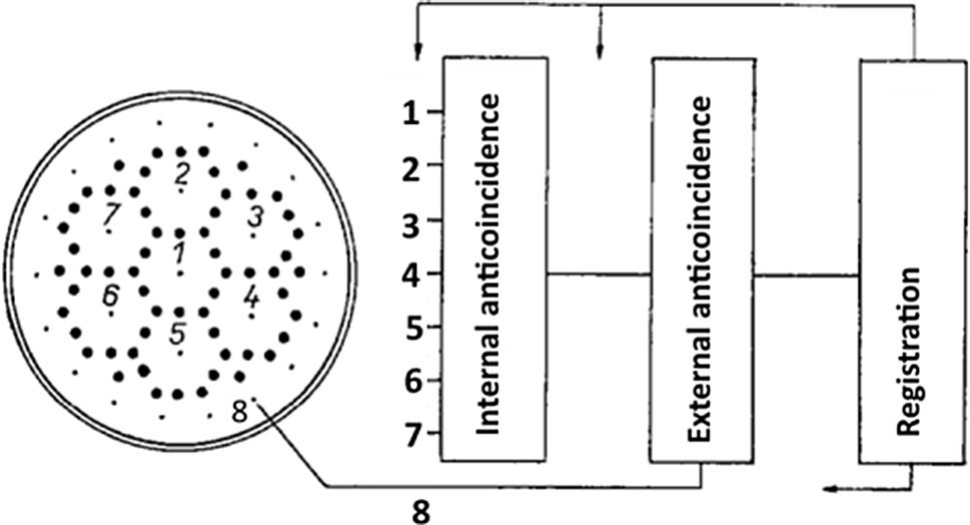
Cross-section of the 7-element proportional counter designed for ^3^H counting (more elements can be added following the project requirements [147, 151])

A simple single wire inner counter has been later replaced by a system of cells forming thus a multielement detector (the Povinec detector) [146–151] (Fig. 2). Such a detection system can be used for tracking beta-electrons from decays of nuclei inside the detector. Except the 3H and 14C activity measurements, the multielement structure was also used for searching of double beta-decay of ^136^Xe in the Gran Sasso underground laboratory (the first experiment carried out in the newly constructed Gran Sasso laboratory [151]). Even a more sophisticated high spatial resolution time projection chambers (possibly inserted into a magnetic field) could be constructed for tracking single beta-electrons [152]. However, these techniques would require large sample volumes. and with the invention of AMS for ^14^C measurements, the radiometric detection systems, both gas, as well as liquid scintillation counters could not compete with AMS measurements. Radiocarbon laboratories with radiometric detectors have, however, significantly contributed to the radiocarbon science, especially in better understanding of the behaviour of radiocarbon in the environment. Most of the high precision ^14^C results (a relative precision of Δ^14^C in modern samples below 0.5%) for the development of the ^14^C calibration curve were obtained using gas proportional and liquid scintillation detectors.

As the maximum energy of beta-electrons emitted by decaying ^3^H and ^14^C nuclei is only 18.6 and 156 keV, respectively, there is a room for decreasing a counter background by discriminating pulses which are above this energy threshold [153]. Using two channel electronics we can register e.g. ^3^H pulses in the first channel and the background pulses above the ^3^H spectrum in the second channel, where about 50% of background pulses originate. Therefore, if we are measuring background in the second channel we may predict its value in the first channel even in the case when we were registering in this channels pulses from ^3^H decays (the Povinec method). We may monitor in this way background variations during long-term measurements (more details can be found in [153]). This is possible to realize either by a multichannel analyser for pulse amplitude evaluation, or by a time–amplitude analysis. Such a registration system has also an advantage in simultaneous registration of pulses from a sample and background, which could help in discriminating false pulses originating either in various electromagnetic disturbances, or by sudden changes in cosmic-ray intensity. As in ^3^H and ^14^C analyses the counting times are typically over a few days, such background monitoring is useful [154]. Recently developed digital multichannel analysers have even better characteristics for long-term monitoring of the detector background, which is frequently used at present in commercial liquid scintillation spectrometers.

### Radiochemical separation methods

**Fig. 3:**
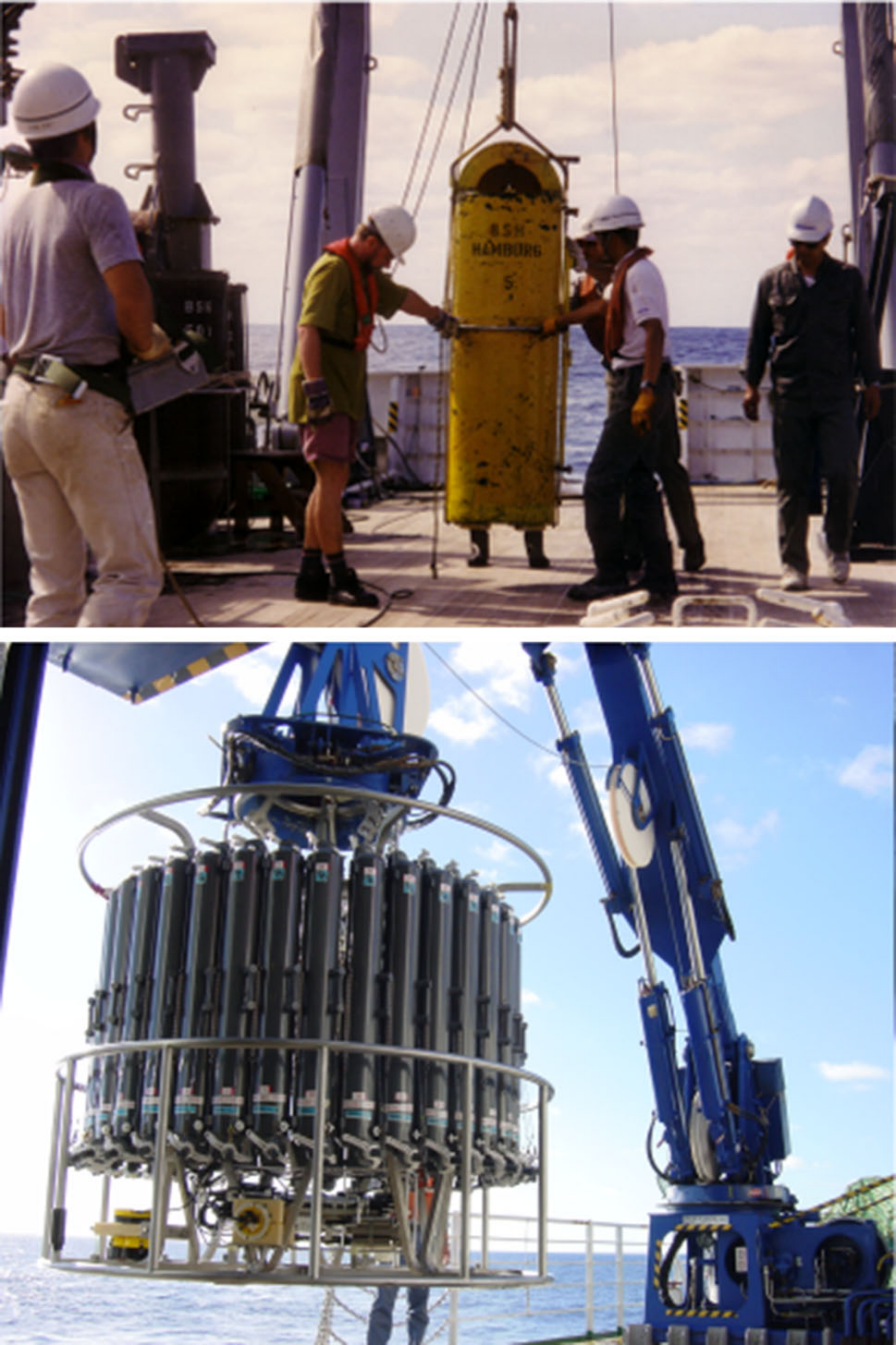
Large-volume (200 L) water samplers (top) compared with the Rosette multisampling system (24 bottles with 20 L volumes) (bottom) operating during deep-water sampling in the Pacific Ocean (IAEA’1997 cruise [22])

Recent developments in radiometric and mass spectrometry technologies have been contributing significantly to new scientific investigations carried out in nuclear and environmental sciences. Sampling techniques have developed from simple devices operating with large volume samplers (from 100 to 500 L, Fig. 3) into the present Rosette multisampling system, and even to robotic systems based on ROVs (Remotely Operating Vehicles). Sophisticated sampling technologies have been accompanied with satellite views of areas for the optimisation of sampling. For example, in the marine environment, where the research work has been heavily depending on the new technologies, we have seen a replacement of time consuming and expensive large volume water sampling (500 L) from several km water depths by Rosette multisampling systems enabling high resolution water sampling of the water column within one or two casts only [1, 3, 4].

There have also been great developments in radiochemical separation methods where we moved from a simple direct/nondestructive analysis of gamma-emitters to complicated radiochemical procedures separating radionuclides of interest from all possible matrices (water, sediment, biota, construction materials, etc.), and finally to robotic systems working on line with computers to minimize human working power in this, traditionally highly time-consuming works. As we have drastically decreased the levels of radionuclides to be analysed, especially in mass spectrometry methods, new problems have arrived, mostly associated with radioactive contamination of chemicals and tracers, and generally with radioactive contamination of laboratories (air, glassware, etc.). Therefore, clean rooms of class 100–1000 have to be used, if ultra-sensitive radionuclide analyses have to be carried out.

**Fig. 4:**
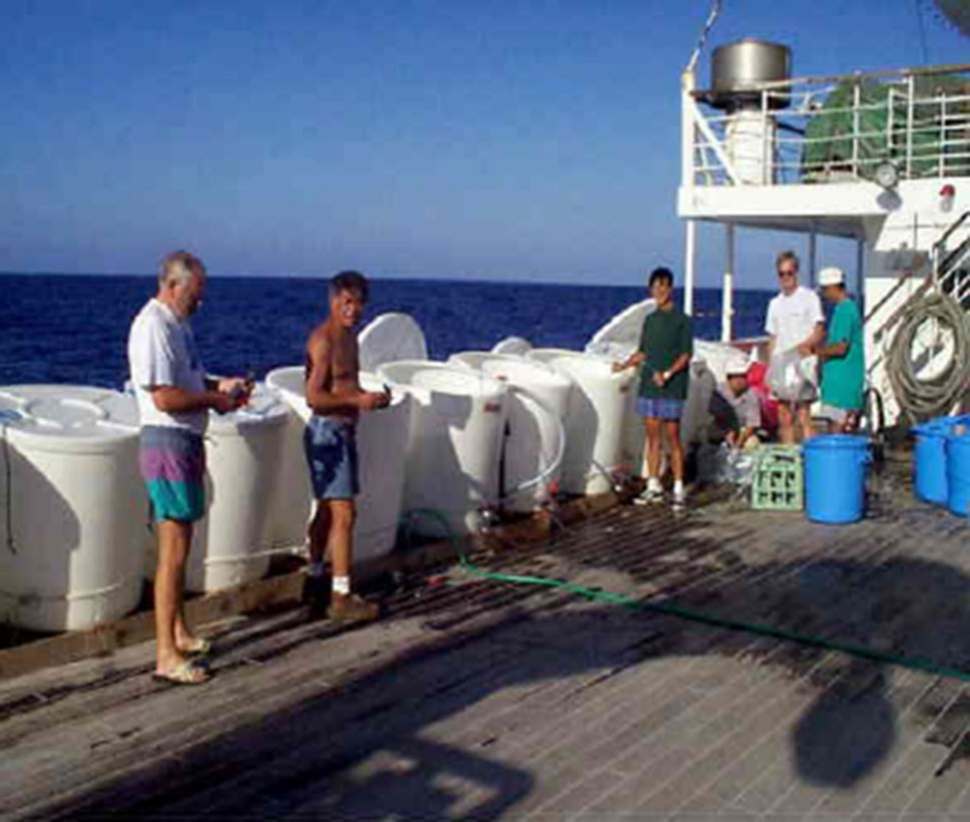
Large volume plastic containers (400 L) on the shipboard for scavenging of ^90^Sr, ^137^Cs, Pu isotopes and ^241^Am from seawater samples (IAEA’1997 cruise [22])

For analysis of all radionuclides of interest, developed radiochemical separation methods represent at present state of the art technologies, specifically focusing on type of the radionuclide, matrices of their occurrence and measuring methods to be applied for analyses. As this is a very widely developed field we shall not go into details, but readers can consult with specialised papers, reviews or monographs to get more information [1, 3, 60]. As a typical example, we present radiochemical procedures to be used for separation of ^90^Sr, ^137^Cs and Pu isotopes from seawater, and their subsequent analyses by beta-, gamma- and alpha-counting, and by AMS/ICPMS. After filtering (0.45 µm mesh) of collected seawater samples, on-board pre-concentration procedures (sequential extraction) were usually carried out to separate ^90^Sr, ^137^Cs and Pu isotopes from the collected seawater samples (MnO_2_ co-precipitation, Fig. 4). ^90^Sr is usually separated by co-precipitation with oxalic acid, and determined using the ^90^Y in-growth method followed by beta-counting in gas or liquid scintillation counters [1, 3, 60]. ^137^Cs is concentrated in seawater samples by adsorption onto AMP (ammonium molybdophosphate) using a method described in detail elsewhere [1, 60], and ^137^Cs activities are determined either directly on AMP solution, or on separated Cs by low-level gamma-spectrometry with high efficiency Ge detectors. Thanks to these new developments it was possible to analyse small volume samples of seawater and thus to reach a high ^137^Cs data density, which allowed to draw a detail picture on the spatial and depth distribution of ^137^Cs in the Indian, Atlantic and Pacific Oceans [33, 107–109].

**Fig. 5:**
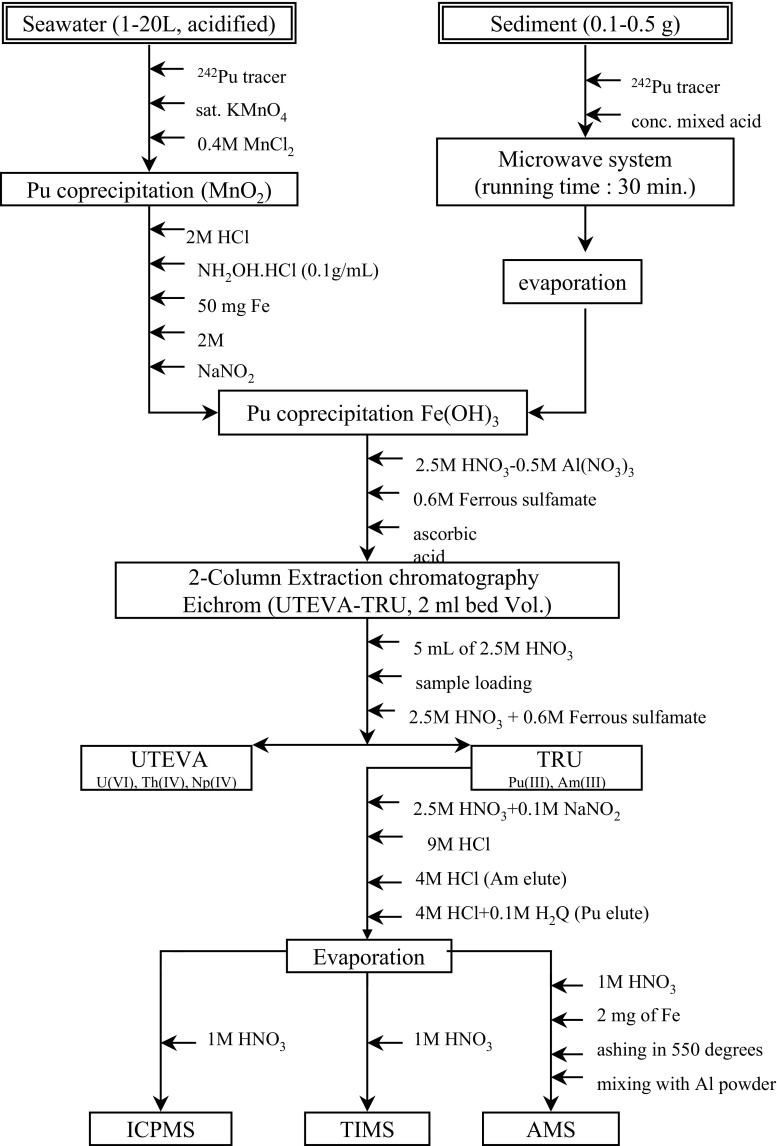
Separation of Pu from seawater and sediments samples and its subsequent analysis by ICPMS, TIMS or AMS

Transuranics are purified using anion-exchange resins and extraction chromatography [110, 111]. The samples are then either electrodeposited on stainless steel disks for alpha-spectrometry or used for ICPMS or AMS [60, 64, 81, 82, 110, 111, 155]. Figure 5 shows as an example a typical flow chart for separation of Pu from seawater and sediment samples and subsequent ICPMS, TIMS or AMS measurements [1, 3].

High efficiency and excellent energy resolution of Ge detectors permit the analyses of gamma-emitters in composite samples selectively and very often non-destructively (e.g., in sea sediments). If such spectrometers can operate at least a few tens of meters underground, or they are protected against penetrating cosmic -ray muons by anticosmic shielding, their performance can be superior in comparison with laboratories located at the surface [1–3, 54, 56, 60]. However, this technique is restricted to gamma-emitters only (e.g., for ^7^Be, ^40^K, ^54^Mn, ^60^Co, ^137^Cs, ^210^Pb, etc.). Other radionuclides frequently found in the marine environment are the pure beta-emitters, like ^3^H, ^14^C, ^32^Si, ^32^P, ^90^Sr, ^241^Pu, etc., where mainly liquid scintillation spectrometry has made great improvements in recent years [112, 113, 156–158]. However, for some of these radionuclides mass spectrometry methods represent a real breakthrough in low-level counting, e.g., “^3^He in-growth” mass spectrometry [159, 160] for ^3^H, or accelerator mass spectrometry for ^14^C [91, 94, 95].

Another important group of radionuclides is represented by alpha-emitters, both natural (like Ra, U and Th isotopes) as well as anthropogenic (like Pu and Am isotopes). These radionuclides have traditionally been analysed by semiconductor alpha-spectrometry. However, there were several limitations, e.g., in sensitivity, resolution and mass of samples used for analysis. Some of them have been partially overcome, e.g., the problems with resolution for analysis of ^239^Pu and ^240^Pu using high resolution alpha-spectrometers with suitable deconvolution software, however, recently especially ICPMS and AMS dominate in analysis of long-lived alpha-emitters in the environment, as these methods eliminate all the above-mentioned problems [2–4, 60, 96].

### Gamma-spectrometers operating in surface laboratories

#### Ge detectors with anticosmic shielding

Availability of large volume Ge detectors has been the most important developments in the radiometric sector. The reasons are in the excellent energy resolution and high efficiency of recently produced Ge detectors (up to about 200%). Muon-induced background becomes dominant for such large volume Ge detectors as the most prominent peaks observed (e.g. annihilation peak, neutron activation peaks) are due to cosmic -ray interactions with Ge detectors [2, 161]. Although the most effective way of increasing the sensitivity of a spectrometer is to increase counting efficiency and the amount of sample to be analysed, frequently, the only possible way is to decrease background of Ge detectors. The background components in a typical low-level Ge detector, not situated deep underground, are cosmic radiation (cosmic muons, neutrons, photons, and material activation products), radioactivity of construction materials, radon and its progenies. For a present-day, carefully designed low-level Ge spectrometer, the dominating background component is cosmic radiation, mainly cosmic-ray muons (Table 2). In a single Ge spectrometer there is no protection against cosmic muons, therefore, a spectrometer with anticosmic shielding will greatly reduce the background. The anticosmic shield can be made of gas or plastic scintillation detectors, which surround the lead/iron/copper shields housing the Ge detector [2, 39, 40, 47, 54, 161]. Another possibility is to use an antiCompton spectrometer, which is a powerful tool for reducing the detector’s background as it combines both anticosmic and antiCompton background suppression [117].

**Table 2:** Detector background components of a low-level Ge detector of 1 kg mass located in a lead shield of 10 cm thick

Background component	Integral counting rate (s^−1^)
Environmental radionuclides	30–300
Muons	0.3–2
Cosmic neutrons	0.03–0.2
Radionuclides in the shield	0.01–0.2
Radon and its daughters	0.01–0.1
Radionuclides in the cryostat	0.003–0.03
Detector activation by cosmic rays	0.0004–0.002

A proper design of a low-level gamma-spectrometer is an important prerequisite for later applications in low-level measurements. High energy cosmic rays can initiate a large number of physical processes leading to background induction. Analytic solutions for describing these processes are not available, therefore statistical technique such as Monte Carlo simulation is necessary. The development of a simulation code for background induction is useful for the optimization of a counting system in respect to its background characteristics. It enables to assert the background before the system is built, and also to perform systematic investigation of the influence of various parameters on the background of the detector. The GEANT code has been used for the simulation of the passage of particles through matter as it meets the requirements for simulation with high-energy muons [2, 42, 161–164]. Monte Carlo predicts that a thickness of 15 cm of lead is the optimum shielding thickness for large volume HPGe detectors situated at sea level or at shallow depths underground. This is much more than usually used 10 cm of lead. If thicker shielding was used in simulations, the background was higher due to interactions of muons with the shield. The simulation also clearly shows that the smallest background is obtained in the smallest shield directly attached to the Ge detector. This has been a surprising result as in previous low-level counting studies opposite recommendations can be found [165]. It is interesting that the background depends only slightly on the shape of the shield if the inner shield dimensions are preserved. Rectangular shields provide only a few percent greater backgrounds than cylindrical ones. The background levels also strongly depend on the internal lining of the shield as the thick layers of low-Z lining increase the detector background (e.g. 1 cm of Cu increase background by factor of two when compared with lead-only shield), explained by smaller self-absorption coefficients for lower-Z materials. Therefore, the shield dimensions should be kept as small as possible (depending on the largest sample size intended for analysis), as the smallest background is obtained in the smallest shield (without any lining, i.e. only with lead walls). For example, it is not worth leaving an extra space in the shield of the well-type detector if samples are only analysed inside the detector well. If the lead X-rays are disturbing, a thin descending Z-lining is superior. It is advisable to design the shield so that the lining can be easily removed. The anticosmic shielding represent an important technology for improving the detection limits of gamma-spectrometers operating at the surface laboratories. A simple plastic scintillation detector placed on the top of the Ge detector which operates as a veto may decrease its background by about a factor of two [161]. The anticosmic shielding is advantageous to use also in shallow underground laboratories operating at depths down to about 100 m w.e. [161].

#### Multidetector gamma-spectrometry

In some applications, a better sensitivity can be obtained by operating a Ge spectrometer with Compton suppression, or as a gamma–gamma coincidence spectrometer. The antiCompton spectrometer combines both the anticosmic and the antiCompton suppression of the background as the principal Ge detector is surrounded by sufficiently large NaI(Tl) or BGO crystals. The Compton suppression factor should reach values around 40, thus improving detection limits significantly [2]. However, high cosmic-muon rejection factors can be reached only if construction materials with negligible radionuclide contamination have been used for the construction of the Ge detector’s cryostat, the surrounding NaI(Tl) detectors and the passive shield. We should also operate the Ge detector in a place with low radon concentration. In specific applications, e.g. analysis of ^210^Pb or ^137^Cs in sea sediments in the presence of a high content of ^40^K it is advantageous to use antiCompton gamma-spectrometer [2, 42, 161, 162]. Due to the relatively high energy of this gamma-emitter (1.46 MeV), radionuclides emitting lower energies of gamma-rays are covered by a Compton continuum from this source. Therefore, the suppression of this continuum (as well as the cosmic-ray induced background) has a great impact on the background of the spectrometer.

**Fig. 6:**
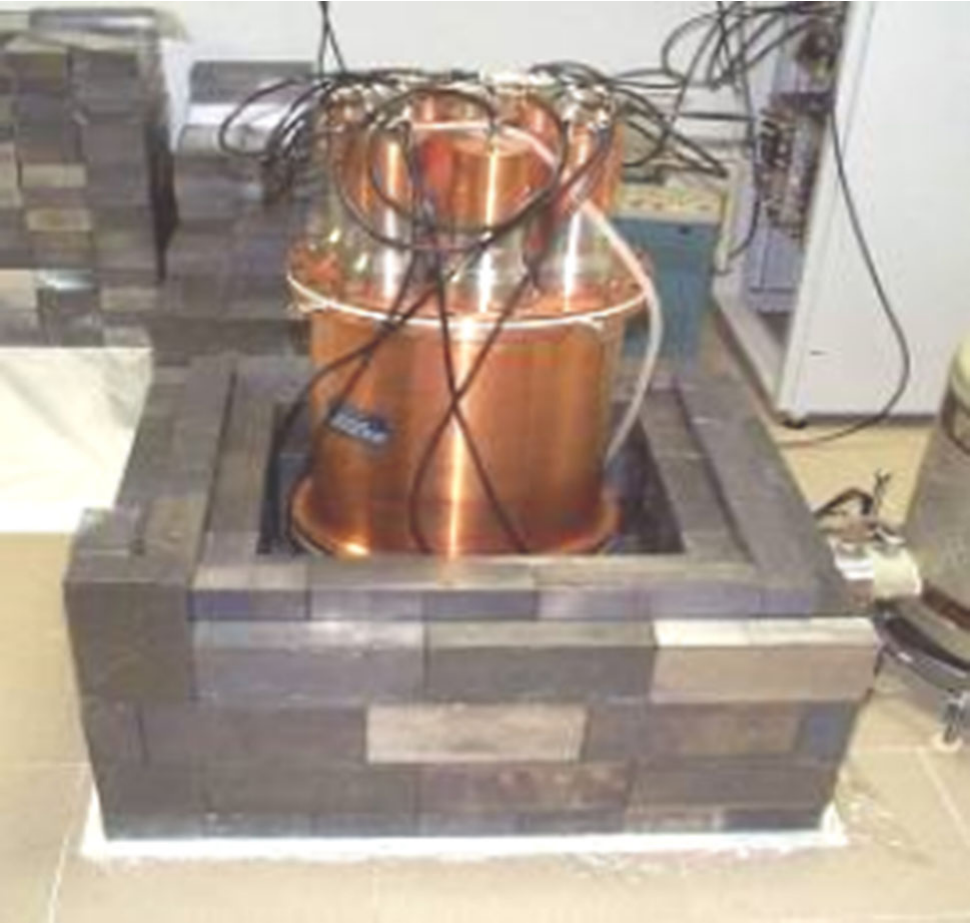
Construction of the anti-Compton gamma-ray spectrometer in the IAEA-EL´s Monaco underground laboratory (100% HPGe detector surrounded by NaI(Tl) detectors in a lead shield)

**Fig. 7:**
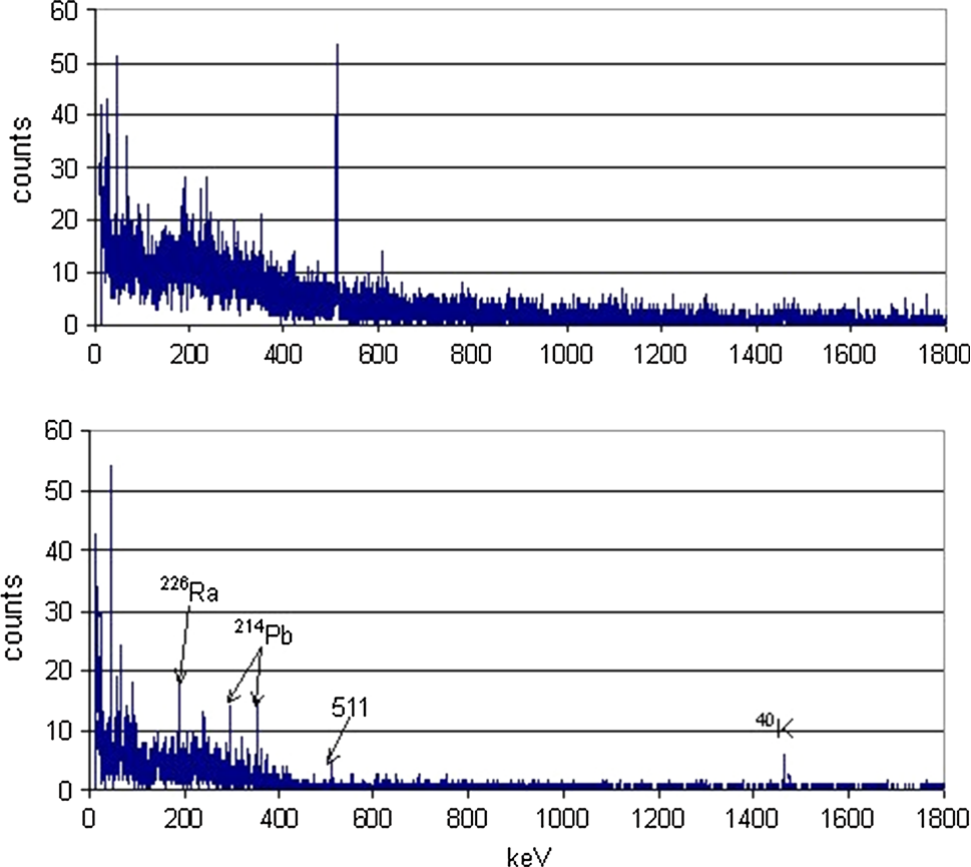
Background spectrum of the antiCompton spectrometer with 100% HPGe detector measured for 60,000 s in the IAEA-EL´s Monaco underground laboratory at 35 m w.e. (top—single spectrum; bottom—anticoincidence spectrum) [2]

AntiCompton gamma-spectrometer with n-type Ge detector (ORTEC, 100% relative efficiency, the peak/Compton ratio 64) has been in operation at the IAEA Monaco laboratory (Fig. 6). The detector arrangement is of a U-type with a preamplifier situated outside of the lead shield, however the FET is mounted on Cu plate connected with cooling finger. The detector cryostat is made of electrolytic copper, the window is made of high purity aluminium. The Ge detector is surrounded by NaI(Tl) shielding. All detectors are housed in a shield made of 10 cm of lead. The background with antiCompton shielding decreased in the energy interval 30–2500 keV by a factor of 5. However, several gamma-lines from natural radionuclides, probably due to a contamination of the detector components and the lead shielding were observed in the Ge-detector background (Fig. 7) [42].

**Fig. 8:**
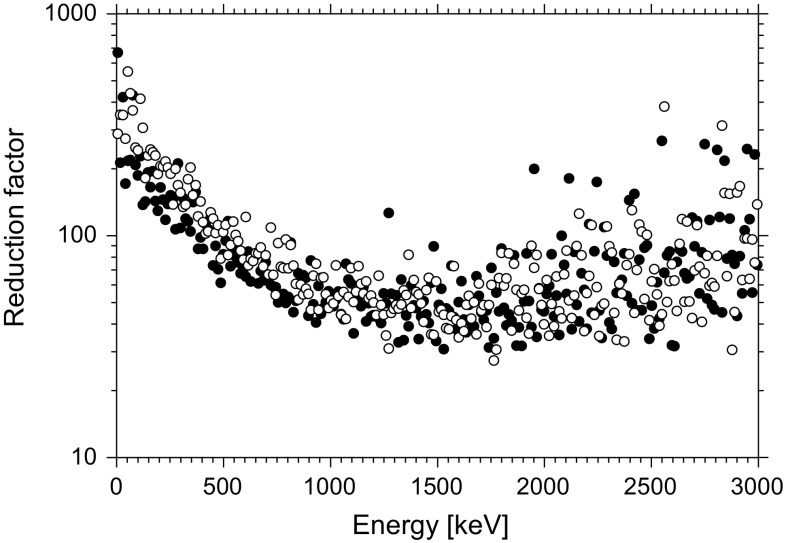
Monte Carlo simulated background reduction factors for an antiCompton gamma-spectrometer operating at sea level (open circles—vertical positions, dots—horizontal positions)

A radioactive contamination of construction materials therefore plays a crucial role in low-level antiCompton spectrometers. As for the operation of a semiconductor detector the diode material, in our case Ge, must be very clean to keep loses of charge collection on the detector electrodes as low as possible, the most probably the detector contamination is caused by a cryostat material. However, in the case of the antiCompton spectrometer the main contaminated components may originate in the NaI(Tl) detectors and photomultipliers. The dominant contaminant there is ^40^K, which is very difficult to remove from the NaI(Tl) detectors. Another possibility would be to use a BGO scintillation detector, which because of higher detection efficiency could have smaller dimensions. However, the radioactive contamination in this case is even worse due to presence of radioactive bismuth and lead. AntiCompton gamma-spectrometers because of contamination problems will not reach therefore a lowest possible background when compared with well-designed single Ge spectrometers. On the other hand, a cosmic-muon rejection factor of at least 40 (at around 1 MeV) is obtained when the antiCompton rejection is operational (Fig. 8) [2]. In such situation, the cosmic-muon background is reduced to such a level that other background components should prevail, like those from the residual contamination of the detector and antiCompton construction materials, or from radon progenies.

Except of single and antiCompton gamma-spectrometer several other detection modes can be used in the coincidence-anticoincidence gamma-spectrometry: (i) Gamma-gamma coincidence mode (e.g. [44, 47]; (ii) triple coincidence mode (e.g. [45, 47]; (iii) summing coincidence mode (e.g. [45, 47]; (iv) beta-gamma coincidence mode (e.g. [47]; (v) beta-gamma-gamma coincidence mode (e.g. [47]; (vi) multidimensional gamma-spectrometer [47, 52, 53]; (vii) gamma–gamma spectrometer for measuring angular distribution of gamma-quanta. Coincidence gamma-spectrometers may decrease a background by about two orders of magnitude, what makes these spectrometers superior for very low-level gamma-spectrometry of positron or cascade gamma-emitters (e.g. ^22^Na, ^26^Al, ^60^Co) [48]. Three-dimensional gamma-spectra (volumetric peaks with better identification) can be obtained from multidimensional spectrometers, which can register both the coincidence and non-coincidence peaks simultaneously. Analysing electronics, if two Ge detectors are used in coincidence, require 8000 × 8000 channels, which, with present state of the art computer electronics, is not a problem. The background of multidimensional gamma-spectrometers can be reduced by about two orders of magnitude.

**Fig. 9:**
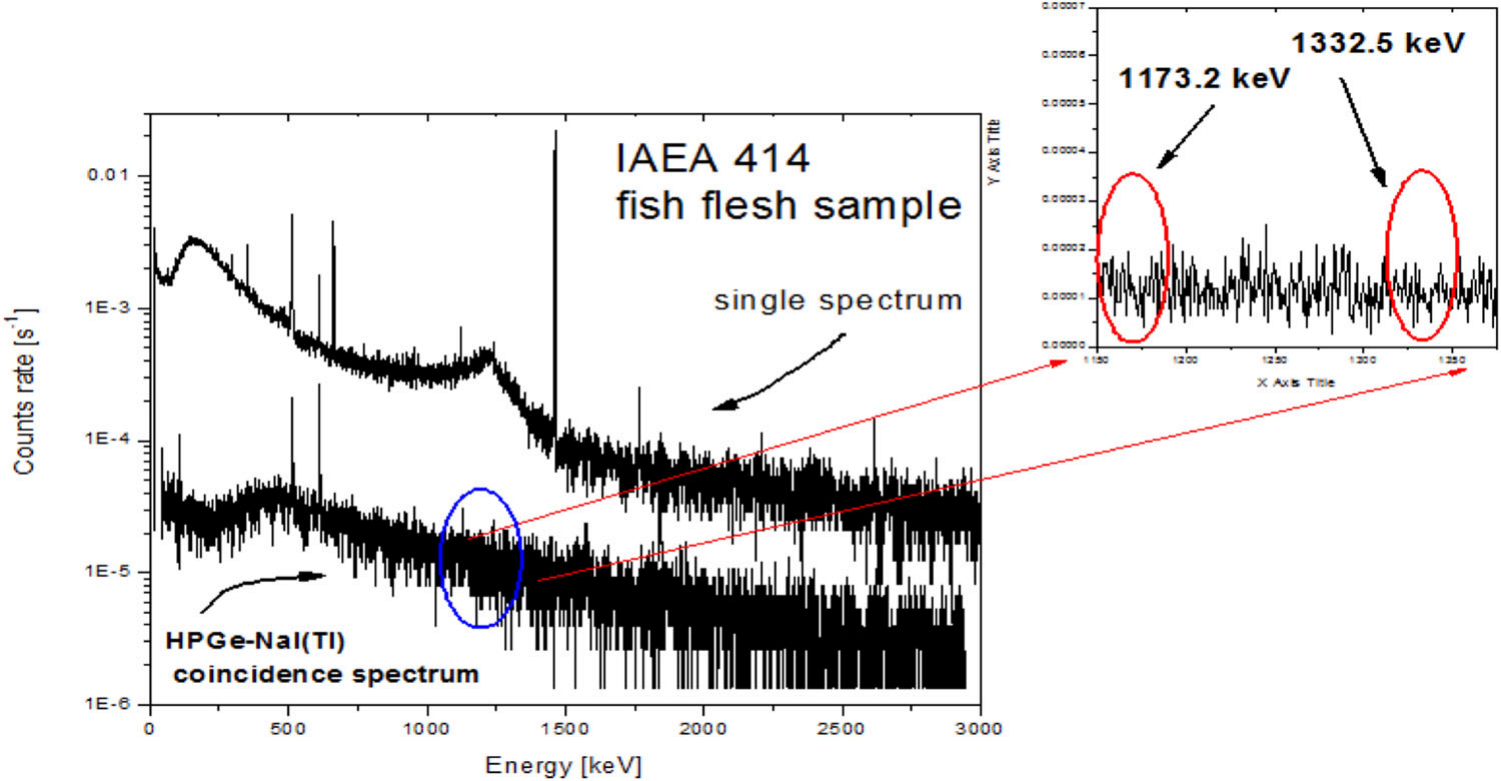
Single and coincidence gamma–gamma spectra of fish flesh sample [50]

Figure 9 compares gamma-spectra obtained by a single Ge detector (70% relative efficiency) with Ge–NaI(Tl) (10 cm in diameter and 10 cm long NaI(Tl) crystal) coincidence spectrometer installed in the Comenius University laboratory [50]. The sensitivity of the coincidence mode is clearly visible, as the background and the Compton continuum from ^40^K has been decreased more than an order of magnitude. The detection limit for ^60^Co in the IAEA-414 reference material (a mixture of Irish Sea and North Sea fish) of 80 mBq kg^−1^ dry weight was obtained.

### Underground gamma-spectrometry laboratories

We have seen that the Monte Carlo simulation tools based on the GEANT proved to be capable to model background characteristics of Ge detectors, and thus to optimise the design of low-level gamma-spectrometry systems. Therefore, the influence of various parameters on the detector background can be studied well in advance, and the cosmic-muon induced background can be estimated before a low-level detector system is constructed. Given the detector set-up, its background spectra induced by cosmic-ray muons can be scaled down by a factor corresponding to the shielding depth. Generally, a radioactive contamination of construction parts of the shield and Ge detectors itself is still dominating factor as the obtained background was always higher than the predicted one by Monte Carlo simulations [174, 175].

#### Shallow-depth laboratories

**Fig. 10:**
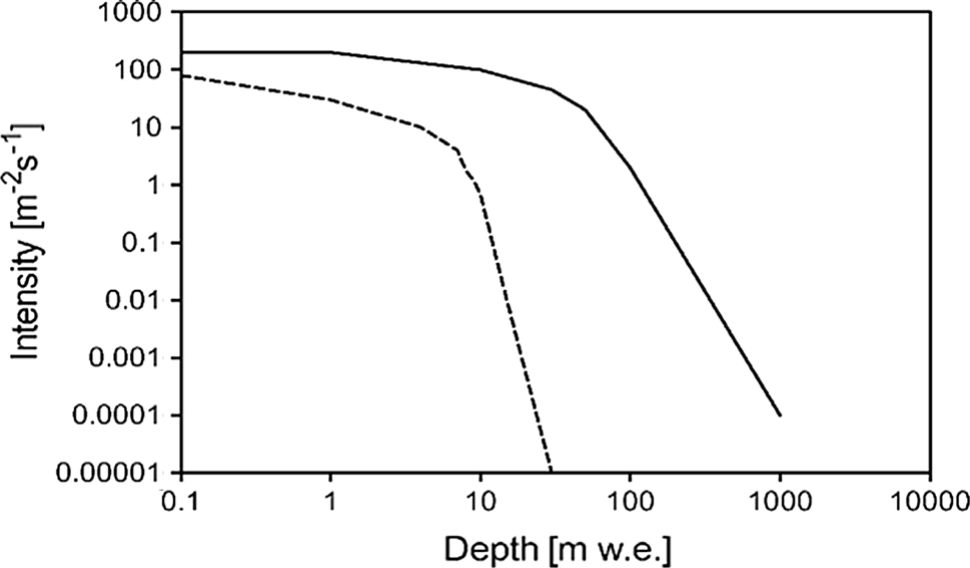
Intensity of secondary cosmic-ray particles with depth underground (the broken line is for neutrons and the full line is for muons)

It has been well known from cosmic-ray physics that fluxes of secondary particles have different behaviour in rocks. While for neutrons the underground depth of 10 m w.e. causes a decrease in their intensity by almost two orders magnitude, for muons an equivalent decrease is reached only at 100 m w.e. (Fig. 10). A low-level Ge spectrometer operating at shallow depth of about 100 m w.e. would benefit therefore at least from a partial suppression of the hard component (muons) of cosmic rays.

**Fig. 11:**
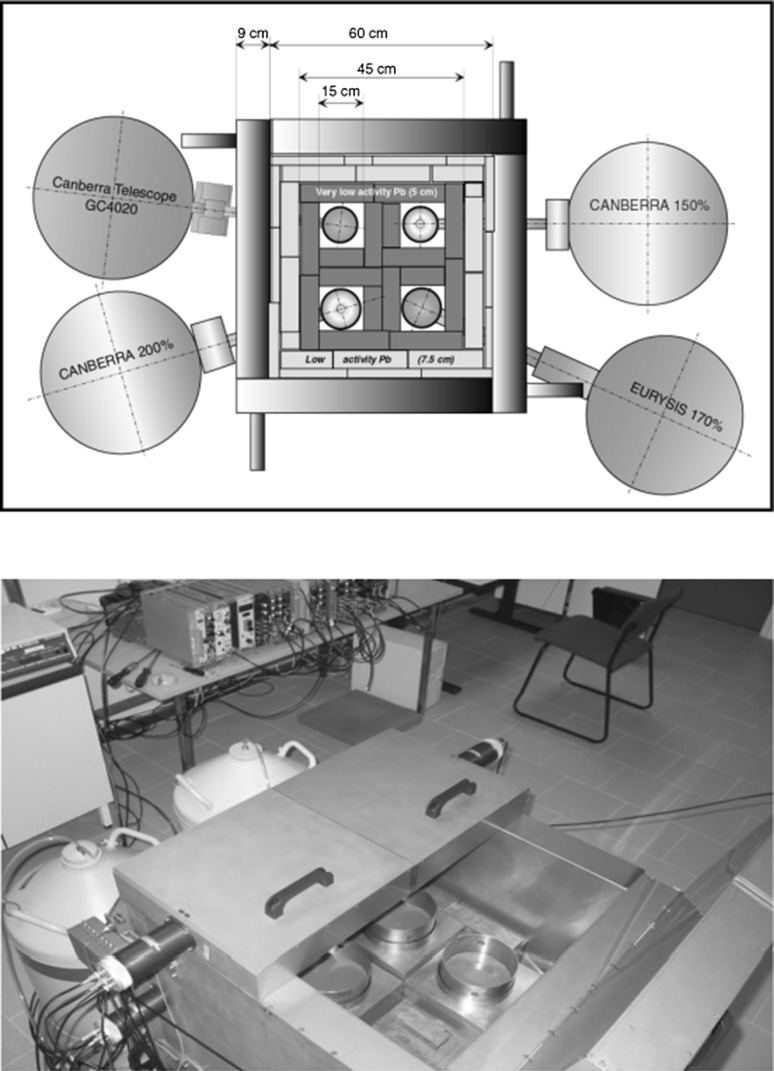
Lead shield with four Ge detectors operating in a shallow underground laboratory in IAEA Monaco with anticosmic shielding made of plastic scintillators [39]

When designing a new underground gamma-spectrometry laboratory, a single Ge spectrometer of about 100% efficiency (especially if the sample size permits the use of a well detector) is the usual choice for majority of environmental applications. We shall illustrate the applications of the Monte Carlo simulations in designing the underground laboratory constructed at the IAEA Monaco [39, 40, 57]. The laboratory is situated in an underground cellar in a car parking area at a depth of 35 m w.e. The laboratory is equipped with a common lead shield housing four large volume Ge detectors (Fig. 11). An anticosmic veto was made of plastic scintillation detectors surrounding the lead shield. Such a novel design, supported by Monte Carlo simulations, when several Ge detectors are placed in the same lead shield with a common anticosmic guard has been used for the first time in low-level gamma-spectrometry. Its big advantages can be summarized as follows: (i) it reduces the mass of expensive lead/copper shield around the Ge crystals; (ii) it reduces the mass of the outer lead/iron shielding; (iii) it reduces the size of the anticosmic shielding protecting the Ge detectors against cosmic-ray muons.

The lead shield housing 4 Ge detectors is made of the outer layer with ordinary lead of 7.5 cm thick and the internal layer of 5 cm thick, made of very low activity lead, which was specially ordered for the underground laboratory (^210^Pb activity is below 0.1 Bq kg^−1^). It should be stressed that this detection system is used only for analysis of low-activity samples. The lead shield is surrounded on all sides and from the top by plastic scintillation sheets 7 cm thick, which are viewed by 5 cm diameter photomultipliers. The Ge detectors (coaxial p-type), specifically designed for low-level gamma-spectrometry in an underground laboratory are of U-type with preamplifiers housed outside of the lead shield, however, the FET is mounted on a Cu plate connected to the cooling finger. Only materials with minimum radionuclide contamination were used for the detector construction. Four types of Ge detectors, with parameters listed in Table 3 are housed in the common lead shield.

**Table 3:** Characteristics of HPGe detectors in the IAEA Monaco underground laboratory

Detector efficiency	100%	150%	170%	200%
Type	Coaxial	Well	Coaxial	Well
Cryostat material	Cu	Cu	Al	Cu
End-cap material	C	Cu	Al	Cu
Ge mass (kg)	2.15	2.50	3.29	4.18
Background at 40–2000 keV (h^−1^ kg^−1^)	290 ± 30	240 ± 20	230 ± 20	200 ± 20
Background at 40–2000 keV with anticosmic veto (h^−1^ kg^−1^)	40 ± 3	27 ± 2	59 ± 4	33 ± 3
Reduction factor	7.2	8.9	3.9	6.2

ORTEC NIM modular electronics have been used for signal processing and data acquisition. During all measurements radon is expelled from the detector chambers by the evaporation of nitrogen from the detector’s Dewar containers, thus keeping stable background during measurements. As the volumes of the detectors differ significantly, it is necessary to compare their background characteristics per kg of Ge. It is interesting to notice that the total detector background per kg of Ge, in the energy window 40–2000 keV, is decreasing with increasing detector volume. However, the background with the anticosmic veto does not follow this rule, but clearly shows a larger contribution of radioactive contamination of construction materials (cryostat plus lead shielding) to its background. The lowest total background with the anticosmic veto was obtained for the 150% efficiency detector, the highest one for the 170% efficiency detector. The 170% efficiency detector have the cryostat made of “pure” aluminium, however, this has been clearly contaminated by U and Th decay products, as well as by ^40^K. It is important therefore that construction materials are carefully checked by the manufacturers for the presence of radionuclides before the detector construction, especially for the detector’s cryostat and its window. However, it has also been a surprise that three detectors with copper cryostats ordered as low background detectors for an underground laboratory from the same company (CANBERRA) had very different background characteristics. The highest background reduction with the anticosmic veto was obtained for the 150% efficiency detector (factor 9), and the lowest for the 170% efficiency detector (factor 4). The 100 and 200% efficiency detectors have the reduction factors within these limits (factors 7 and 6, respectively). The obtained background reduction factors are considerably lower than we would expect from Monte Carlo simulations, which may be due to several reasons: (i) Leakage of muons through the anticosmic shielding, either due to the shielding geometry or energy off-set in the scintillation detectors; (ii) cosmic-ray secondaries produced by muons passing the lead shield which were not discriminated by the anticoincidence circuit. A contamination of the detector’s window may also be important, e.g. it may be advantage to made it from thin copper if the threshold energy need not to be very low. If a low energy window is required, an ultra-clean aluminium or a carbon fibre may be a good choice. There are other construction materials that could affect the detector background as well. Preamplifier is usually situated outside of the lead shield, however the FET transistor is connected directly with the Ge diode. The detector holder, copper cooling finger and soldering contacts may be therefore crucial for obtaining a low detector background.

Because of shallow operating depth, the annihilation peak at 511 keV is still dominant in background gamma-spectra, as a result of annihilation of electrons and positrons in the shield and in the Ge detector, which are products of the interaction of secondary cosmic rays with materials surrounding the detector. All these drawbacks would be overcome in deep underground laboratories where fluxes of cosmic-ray secondaries are negligible (Fig. 10).

**Fig. 12:**
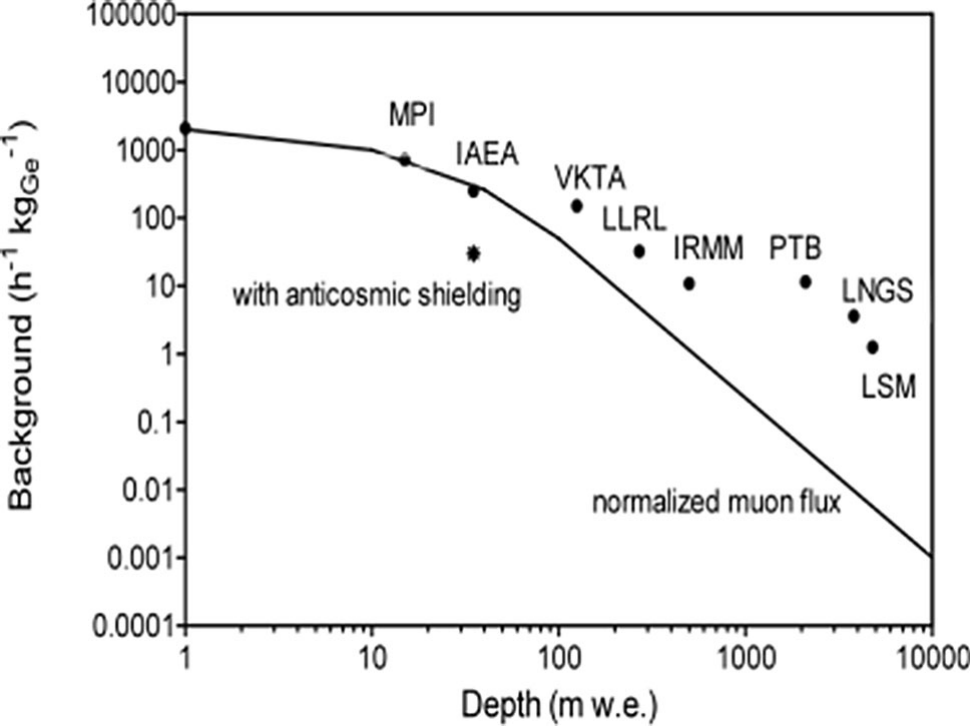
Comparison of gamma-spectrometers backgrounds operating at different depths [2]

The background of the described underground Ge detectors (operating only at 35 m w.e. but with anticosmic veto) when compared with other underground laboratories (normalized to the Ge detector mass) is similar to underground laboratories operating with a passive shielding at 250 m w.e. depth [166–168]. This is even better seen in Fig. 12 where integral backgrounds of Ge detectors divided by the mass of the Ge crystal, operating in different underground laboratories are compared [169]. Therefore, an anticosmic shielding in an underground laboratory operating at a shallow depth is extremely important for reducing the detector background, and should be widely used. For example, in the case of analysis of ^137^Cs in seawater samples it has been possible to decrease the sample volume by about a factor of 10, which greatly reduces sampling time. Another advantage is that the same seawater volumes could be used for gamma-spectrometry as well as for mass spectrometry measurements, therefore large samples of about 200 L are not required [1, 2, 107–109].

### Deep-underground laboratories

The most important recent breakthrough in the radiometrics technologies is represented by operation of gamma-spectrometers in deep underground laboratories. However, we have already noticed (Fig. 12) that for the present state of the art Ge low background detectors it is very difficult to fully utilize for further background reductions depths below 1000 m w.e. Although in deeper laboratories the muon flux is much weaker, it does not improve anymore background characteristics of Ge detectors. We believe that a further reduction in the detector background would be possible only with a new generation of Ge detectors, specially designed (and produced) for deep underground laboratories. Special arrangements must be also made how to decrease a radon contribution to the detector background, especially if frequent changes of samples in the detection system are required.

Cosmic-ray contributions to the background of a Ge detector operating deep underground (deeper than 1000 m w.e.) became marginal as the muon flux is effectively decreased by several orders of magnitude to negligible levels: (i) In the Gran Sasso National Laboratory, GSNL, Italy (depth 3800 m w.e.) the muon flux is 3.4 × 10^−4^ m^−2^ s^−1^ [170]; (ii) in the Laboratoire Souterrain de Modane, LSM, France (depth 4800 m w.e.) the muon flux is 6.2 × 10^−5^ m^−2^s^−1^ [171]; (iii) in the Sudbury Neutrino Observatory, SNOLAB, Canada (depth 6010 m w.e.) the muon flux is 3.1 × 10^−5^ m^−2^s^−1^ [172]. Therefore, the main background components will originate from radionuclide contamination of the construction parts of the detector, a passive shielding and surrounding rocks. Rocks with high uranium and thorium content should be therefore avoided for location of underground laboratories, not only because of large gamma-fluxes from their daughter products, production of radon and thoron, but also due to production of neutrons in (alpha, n) reactions. Table 4 shows that from this point of view the best rock material would be limestone and sandstone.

**Table 4:** Calculated neutron fluxes from fission and (α,n) reactions in different rocks

Rock type	Neutron flux (kg^−1^ day^−1^)
Granite	60
Salt	20
Sandstone	5
Limestone	4

**Fig. 13:**
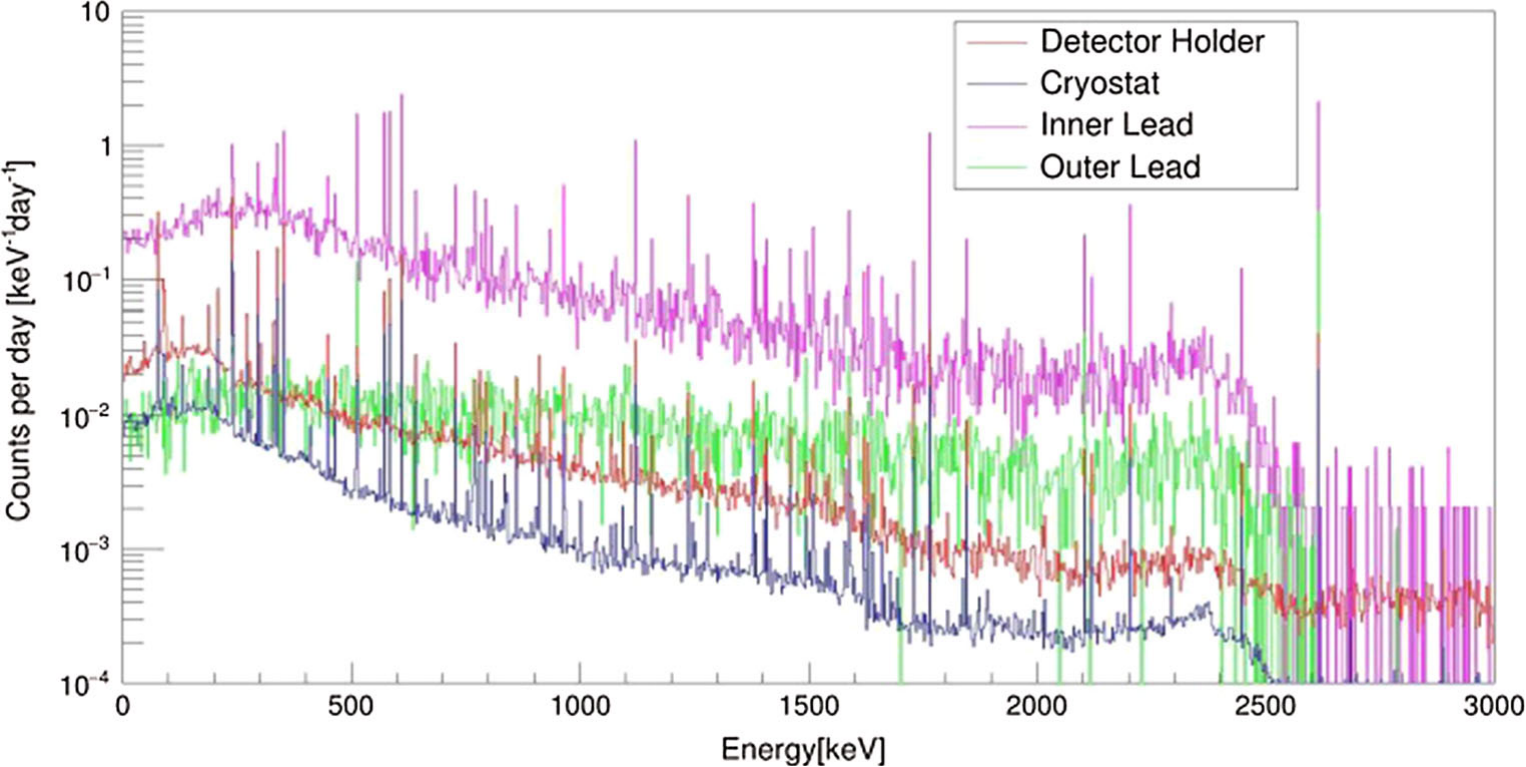
Monte Carlo simulated background gamma-ray spectra for the Modane underground laboratory (scaled to 1 mBq kg^−1^) [175]

**Fig. 14:**
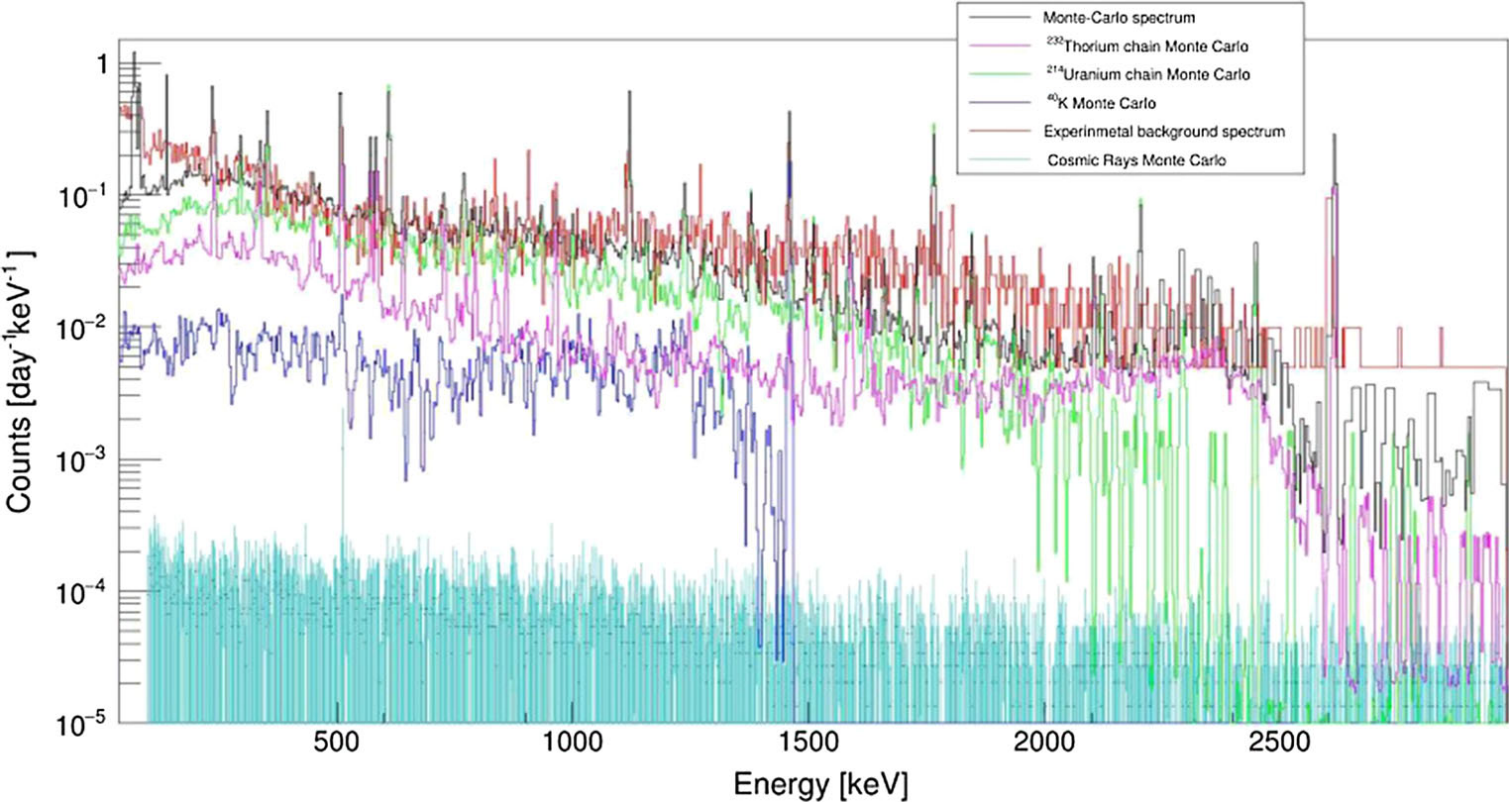
Comparison of Monte Carlo simulated background gamma-spectra with contributions from different radionuclides with experimental spectrum measured in the Modane underground laboratory [175]

Monte Carlo simulations of background characteristics of Ge spectrometers operating deep underground were therefore carried out to better understand the background sources [173–175]. Monte Carlo simulated gamma-ray spectra originating from different parts of the Ge spectrometer operating in the Gran Sasso and Modane underground laboratories were studied [174, 175]. We shall discuss in detail results for the Modane laboratory (depth of 4800 m w.e., Ge crystal of 160% efficiency, cryostat made of pure aluminium, inner (ancient lead) and the outer lead shielding). Figure 13 shows gamma-ray spectra simulated from different places outside of the Ge crystal, normalized to 1 mBq kg^−1^. The biggest background contribution is from the inner lead (although this is a relatively radiopure material), followed by the outer lead, the copper detector holder, and the aluminium cryostat. It is clear that the mass of the contaminated material and its distance from the Ge crystal (absorption properties) made the dominant contribution to the background (as normalized to 1 mBq kg^−1^). The Monte Carlo simulated gamma-spectra for different radionuclide contaminants (normalized to the main peaks) follow the measured spectrum (Fig. 14). The lowest background continuum is simulated from ^40^K, followed by the ^232^Th chain. On the other hand, the ^238^U chain has a main contribution to the detector background, except for the energies above 2000 keV, where the ^232^Th chain dominates due to ^208^Tl contribution at 2615 keV. The Monte Carlo simulated cosmic-ray background gamma-spectrum is, however, by about three orders of magnitude lower than the measured one.

**Fig. 15:**
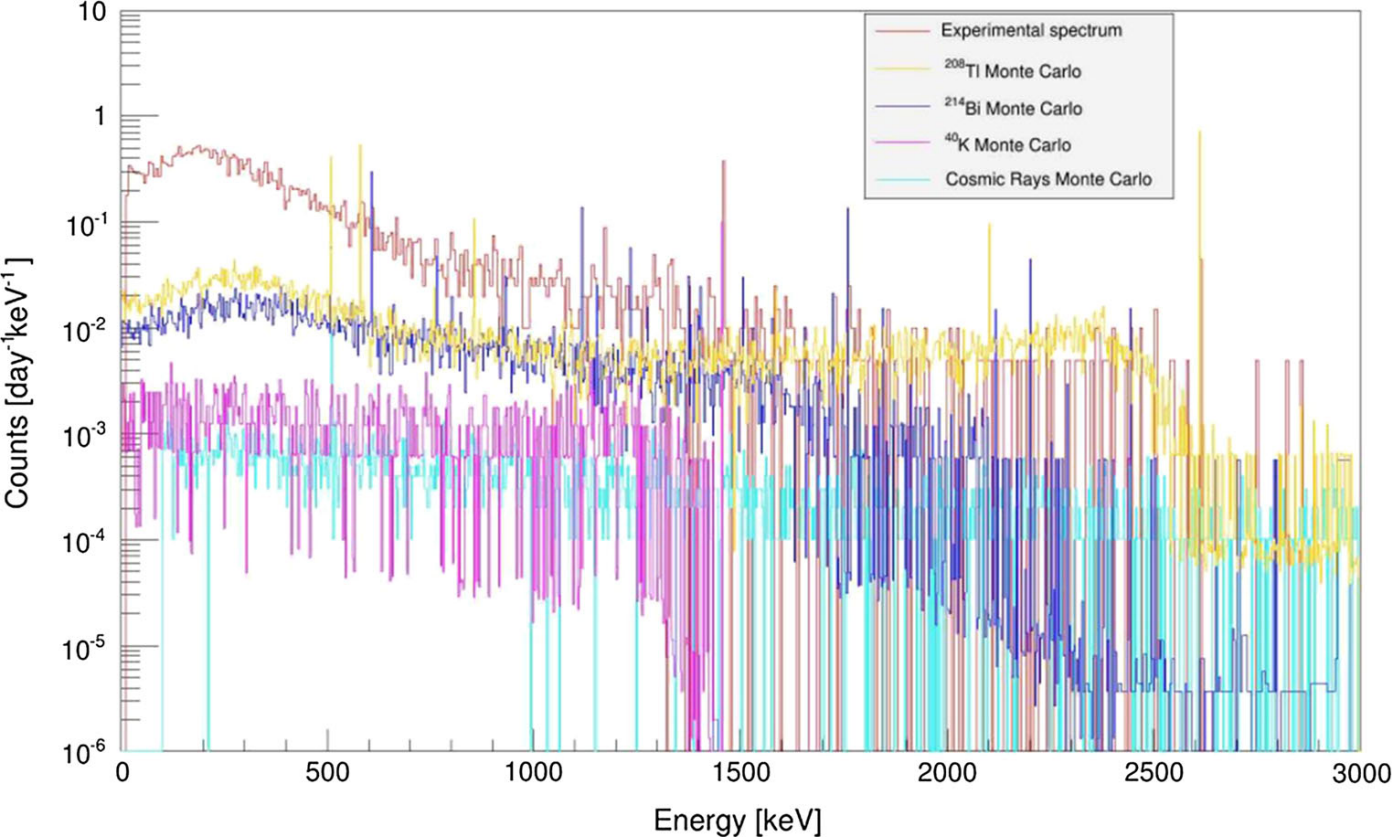
Monte Carlo simulated Ge background contaminant gamma-spectra compared with spectrum measured in the Gran Sasso underground laboratory [174]

Similar results were obtained for Ge gamma-spectrometer (with active volume of the detector of 465 cm^3^) operating in the Gran Sasso underground laboratory at the depth of 3800 m w.e. as documented in Fig. 15 [174]. The Monte Carlo simulated ^214^Bi and ^208^Tl contributions are very similar for energies below 1500 keV, however, at higher energies the dominant contaminant is ^208^Tl (mainly because of the peak at 2615 keV and its Compton continuum). A comparison of the experimental background gamma-spectrum of the Ge detector with Monte Carlo simulations clearly shows that the experimental spectrum was by about two orders of magnitude higher than the simulated one. The difference is again due to the presence of natural radioactivity in construction parts placed around the Ge detector (cryostat, window, electronics, connectors, cables) as well as its surroundings (lead and copper shielding, laboratory walls).

Both Ge-detector background studies carried out for the Modane and Gran Sasso underground laboratories have confirmed that radionuclide contamination of construction materials surrounding Ge crystals makes serious limitations for future improvements in gamma-spectrometers sensitivities. Better choice of radiopure construction materials should be carried out otherwise the advantages of deep underground laboratories will be lost for ultra-sensitive radioactivity measurements. The obtained results also indicate that it would be rather difficult to overcome a detection limit of underground Ge gamma-spectrometers (1 µBq kg^−1^), and thus to use this technique more effectively for ultra-sensitive radiopurity measurements of construction materials as the same radionuclides for which we are searching are the main background constituents even of the ultra-sensitive Ge gamma-spectrometers operating deep underground. Therefore, for U and Th detection limits below 1 µBq kg^−1^ new mass spectrometry technologies may be a solution.

## Developments in mass spectrometry technologies

As we already mentioned, the most important recent developments in the radioanalytical technologies for analysis of long-lived radionuclides have been applications of mass spectrometric systems, especially the AMS and the ICPMS. When using measurement techniques with the extreme sensitivity at levels below 10^−15^ g per sample, it is very important to minimize the effects of airborne dust particles, reagents, glassware, etc., which can all contribute significantly to the sample blank, and as a result they will modify analytical detection limits which are actually defined by the contamination of the blank sample. For this reason, a successful operation of mass spectrometric systems requires more careful sampling, very clean chemical processing and a higher level of instrumental expertise than in any other radioanalytical methods. Because of limited space available we shall focus in this paper only on ICPMS and AMS technologies.

### Inductively coupled plasma mass spectrometry (ICPMS)

ICPMS has developed into a powerful technique for the analysis of elements, stable and long-lived radioactive isotopes in the environment. With the introduction of the present state of the art high resolution ICPMS machines, this technique competes with TIMS in many respects. The principal advantages of ICPMS are its capability to determine long-lived radioisotopes of metallic elements down to fg levels, to analyse aqueous samples directly and rapidly (in a few minutes) at the low cost per analysis and with small sample size. However, ICPMS is not free of matrix and isotopic effects, therefore careful purification procedures (using e.g. extraction chromatography) are required. A steady increase over the past decade in radioanalytical applications using ICMPS has resulted in a decrease in both the price of instruments and detection limits. New generation of sector field instruments with double-focusing and even multi-collector systems have improved sensitivity (by about an order of magnitude) and precision over traditional quadrupole machines [81, 85, 123]. ICPMS has been used in both higher-resolution and lower-resolution modes. The higher-resolution mode has the advantage of addressing polyatomic interferences, although it cannot solve all the problems with isobaric interferences, which may be caused by incomplete separation chemistry. On the other hand, maximum sensitivity can be reached in the lower-resolution mode. Thus, a combination of the two modes appears to be the best compromise for reaching maximum sensitivity and controlling interferences. The higher count rates under lower-resolution mode give better analytical peaks with lower uncertainties and optimal data quality. Analytes with a relatively strong probability of polyatomic interferences on the isotopes of interest (e.g. ^238^U which produces a hydride peak that would interfere with ^239^Pu) should always be scanned. There are problems, however, with the relatively poor abundance sensitivity of sector field ICPMS in the measurement of isotopes with one mass below an abundance peak (e.g. ^237^Np in the presence of high ^238^U), and two mass units below (e.g. ^230^Th in the presence of ^232^Th, or ^236^U in the presence of high ^238^U content). Even when sample matrices are reasonably clean, and care has been taken to minimize oxides during tuning, measurements made near detection limits are sensitive to overestimations due to polyatomic interferences. The use of chromatographic resins [97, 110, 111] has been found to be a suitable technique for processing small volume samples, removing possible interferences by additional cleaning, as well as for cleaning leached plutonium samples electrodeposited on stainless steel disks, previously analysed by alpha-spectrometry.

**Fig. 16:**
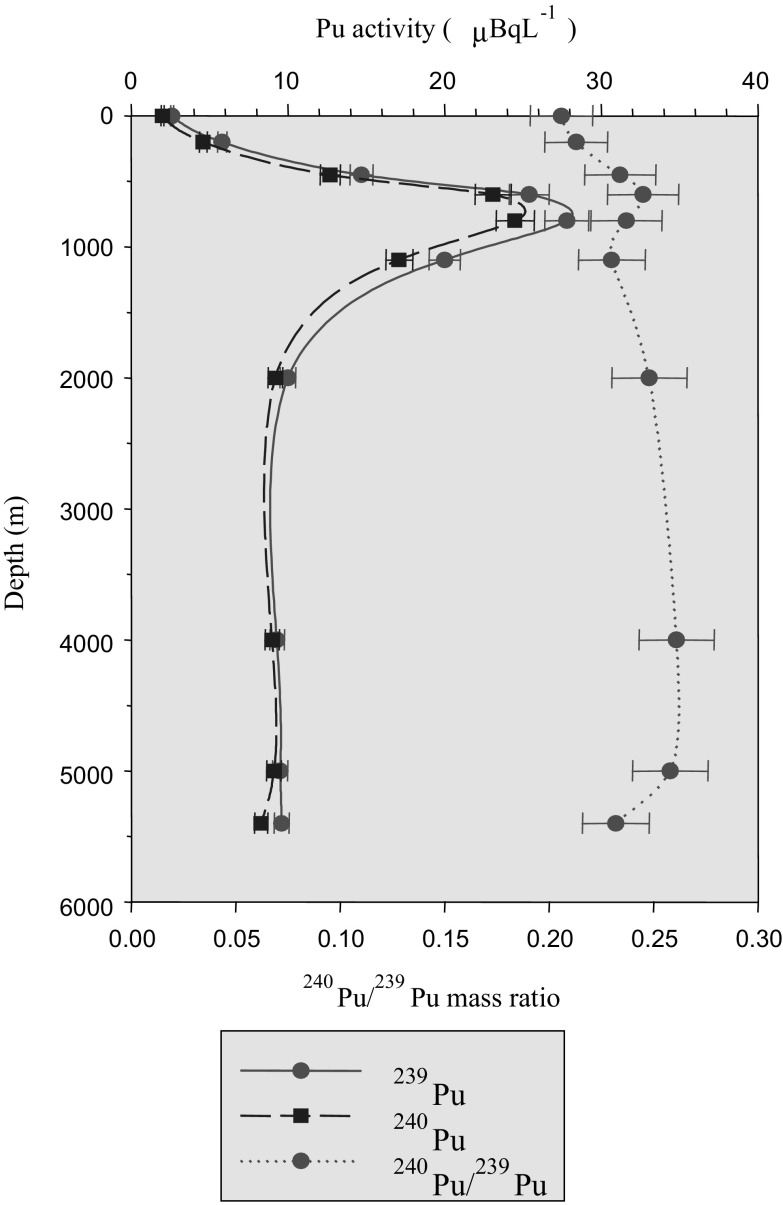
Plutonium isotope seawater profiles in the North-West Pacific Ocean measured by ICPMS [22]

Although molecular, isobaric and isotopic interferences remain crucial for successful operation of ICPMS, this technique has a large potential for automation by direct coupling with new generation of chromatography instruments. With the introduction of high resolution and high sensitive ICPMS it has been possible to analyse some of the long-lived radionuclides like ^99^Tc, ^129^I, ^236^U, ^239^Pu and ^240^Pu at very low levels [84, 85]. This was especially advantageous for reporting separate data for ^239^Pu and ^240^Pu, and using their ratio for tracing the origin of plutonium in the environment. ^238^Pu is difficult to analyse by ICPMS as tracers of ^238^U (even after careful separation) may be present in the sample. ICPMS has also been widely applied on analysis of long-lived ^99^Tc and ^129^I in seawater and seaweeds [85]. Figure 16 shows typical example of ^239^Pu and ^240^Pu water profile activity concentrations (together with ^240^Pu/^239^Pu mass ratios) taken close to the Enewetak Atoll [22]. The medium depth peak, located at 500 m water depth, is clearly visible for both radionuclides, as well as the higher concentrations measured in the bottom sample. The ^240^Pu/^239^Pu ratio is higher than expected from global fallout (0.186), indicating the influence of high-yield nuclear weapons tests conducted in Bikini and Enewetak atolls on Pu concentrations in the water column of the western North Pacific subtropical gyre [1, 4, 19, 20]. ICPMS has also been effectively applied in certification of reference materials. As an independent method (important for production of certified reference materials) has been used for measurement of U, Th, Pu isotopes in terrestrial and marine environment (sediment, seawater, biota).

### Accelerator mass spectrometry (AMS)

AMS represents the most important breakthrough in the analysis of some of the long-lived radionuclides. Development of dedicated tandem accelerators for AMS, firstly for ^14^C, and then for other radionuclides as well (^3^H, ^10^Be, ^26^Al, ^32^Si, ^36^Cl, ^39^Ar, ^41^Ca, ^53^Mn, ^99^Tc, ^129^I, ^135^Cs, ^237^Np, U and Pu isotopes) expanded AMS applications in all sciences dealing with radionuclide analyses. As in tandem accelerators only negative ions can be used for acceleration, the AMS technique can only be applied for those elements (the great majority) forming negative ions, however, recently there has been research going on using positive ions as well. The most important recent developments in the AMS sector are based on small AMS machines (200–500 kV only) which eliminated the need for a costly pressure tank. This interesting design is a new competitor to larger AMS machines for ^14^C dating. However, as the operating terminal voltage used for AMS decreases, the technical engineering difficulties increase. The small AMS machines, especially those operating at 1 MV have also a considerable potential for applications for other already mentioned long-lived radionuclides. We may summarize that the AMS has become the most sensitive technique at present for the analysis of long-lived radionuclides [91–96].

**Fig. 17:**
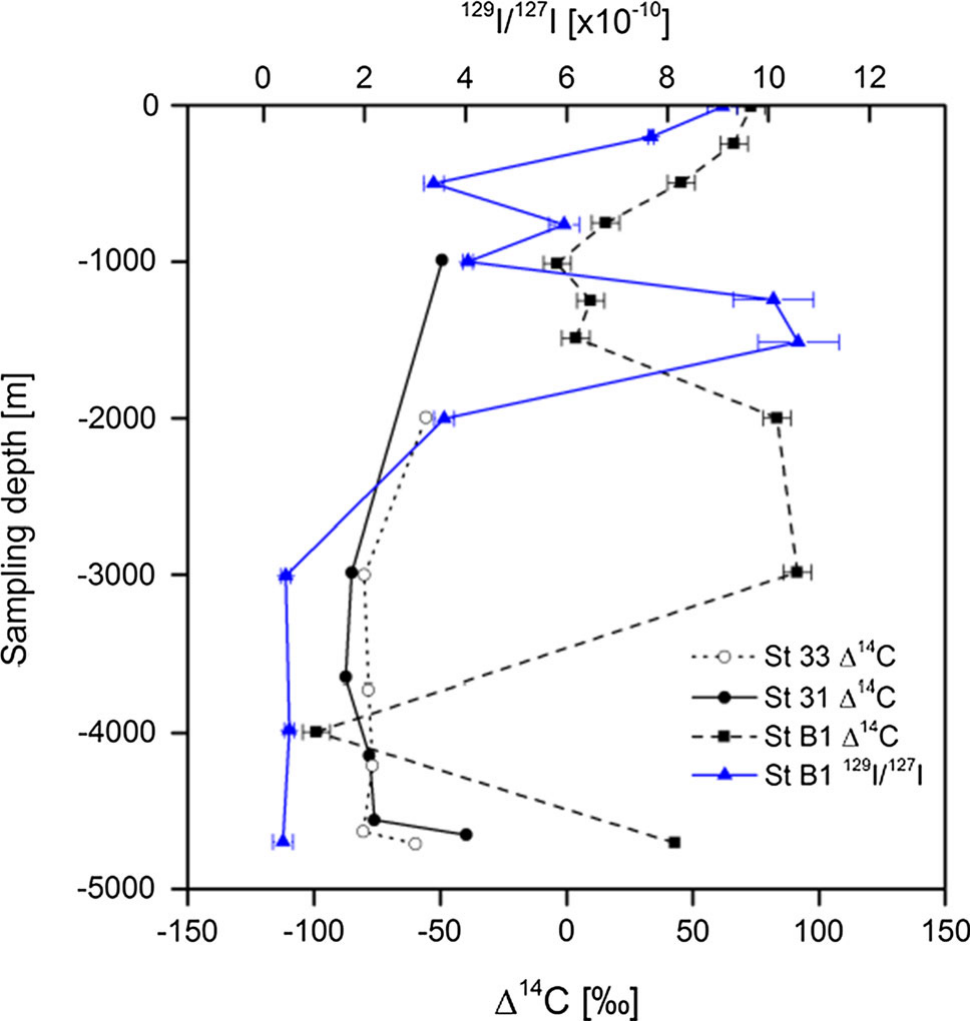
^14^C and ^129^I/^127^I water profiles of NE Atlantic Ocean [93]

We shall illustrate the high analytical sensitivity offered by AMS in tracer amount studies of ^14^C and ^129^I in seawater around dumped radioactive wastes in the NE Atlantic dumping sites. The ^14^C results depicted in Fig. 17 show a remarkable peak at medium depths (between 2000 and 3000 m) which is due to the subduction mode water regime when water masses from the surface are transported to medium depths [93]. The ^129^I/^127^I ratio measured in the same set of seawater samples also showed a medium depth peak, although it has moved to 1250–1500 m water depth. The higher bottom ∆^14^C values may indicate a leakage from the wastes, as the ^129^I/^127^I ratios below 3000 m have shown constant values.

In the framework of marine radioactivity assessment of radioactive waste dumping sites (Atlantic, Arctic and Pacific Oceans), radiological assessment of nuclear bomb tests sites (French and USA testing sites in the Pacific Ocean), as well as a wide range of marine radioactivity surveys we established background levels of key radionuclides (^3^H, ^14^C, ^90^Sr, ^129^I, ^137^Cs, U, Pu and Am isotopes) in the World Ocean so any recent releases of these radionuclides would be easily recognized over the global fallout background [1–4, 93, 99, 176–179].

## A comparison of radiometric and mass spectrometry techniques

In the case of ^3^H there is not an alternative method to in-growth ^3^He mass spectrometry method for ^3^H analyses down to 0.01 TU. Similarly, for ^14^C and ^129^I analysis of environmental samples, especially for water column investigations, the AMS technique is the dominant one. In the case of analysis of ^137^Cs in water column samples the underground gamma-ray spectrometry is the suitable technique which can produce high density data sets. A comparison of Pu results obtained by AMS, ICPMS and semiconductor alpha-spectrometry (SAS) shows that a reasonably good agreement (within quoted uncertainties) for wide range of activities and different sample matrices analysed has been obtained for all three methods. From the point of view of sensitivity, AMS and ICPMS have detection limits at least by a factor of 10 lower than SAS. Sample chemistry, injection and the physics of the analysis make the ICPMS a simpler technique than the AMS, however, because of a higher sensitivity to possible interferences (e.g. a production of hydrates) the AMS is the most suitable technique for analysis of ^239^Pu and ^240^Pu in environmental samples. A comparison of Pu results obtained by ICPMS, AMS and SAS is presented in Table 5. It can be seen that a reasonably good agreement has been obtained between all three methods. The relative precision of Pu results as obtained until now by SAS, AMS and ICP-MS was 5, 10 and 7%, respectively (for ^239,240^Pu).

**Table 5:** A comparison of results of analysis of Pu and U isotopes in IAEA reference materials

Method	^238^Pu Bq kg^−1^	^239^Pu Bq kg^−1^	^240^Pu Bq kg^−1^	^239,240^Pu Bq kg^−1^	^241^Pu Bq kg^−1^	^242^Pu Bq kg^−1^	^238^U Bq kg^−^	^236^U Bq kg^−1^
IAEA-134 Irish Sea cockle flesh								
SAS	3.0 ± 0.2			16.3 ± 0.8				
ICPMS		9.8 ± 0.8	7.7 ± 0.6	17.5 ± 1.0				
IAEA-135 Irish Sea sediment								
SAS	42 ± 1			222 ± 8				
AMS		129 ± 13	92 ± 9	221 ± 16				(11.2 ± 2.2) 10^−3^
ICPMS		127 ± 10	98 ± 8	221 ± 13	3970 ± 200	0.051 ± 0.001		(10.3 ± 3.0) 10^−3^
LSS					3000 ± 650	0.056 ± 0.006		
IAEA381 Irish Sea water								
SAS	3.3 ± 2			14.0 ± 1.0			0.042 ± 0.004	
AMS		8.2 ± 0.3	7.0 ± 0.2	15.7 ± 0.4		0.0046 ± 0.0005		(0.019 ± 0.003) 10^−3^
ICPMS		8.1 ± 0.8	7.0 ± 0.7	15.1 ± 1.1		0.0047 ± 0.0002		(0.023 ± 0.006) 10^−3^
LSS					200 ± 60			
IAEA-384 Fangataufa lagoon sediment								
SAS	40 ± 2			111 ± 3			35.3 ± 1.7	
AMS		109 ± 11	14 ± 1	123 ± 11		2.5 ± 0.4		(8.4 ± 1.7) 10^−6^
ICPMS		102 ± 8	18 ± 2	120 ± 8	240 ± 20	1.9 ± 0.5		
LSS					100 ± 70			
IAEA-385 Irish Sea sediment								
SAS	0.45 ± 0.03			2.94 ± 0.09			29 ± 1	
AMS		1.79 ± 0.28	1.15 ± 0.14					< 0.0001
LSS					30 ± 2			
IAEA-414 Irish and North Sea fish								
SAS	0.025 ± 0.002			0.120 ± 0.005		0.044 ± 0.004	1.11 ± 0.07	
AMS		0.087 ± 0.003	0.053 ± 0.005	0.14 ± 0.01		0.068 ± 0.010		(0.024 ± 0.007) 10^−3^
0 ICPMS		0.063 ± 0.007	0.046 ± 0.005	0.11 ± 0.02	2.7 ± 0.6			

A general comparison of radiometric and mass spectrometry techniques is presented in Table 6. It can be seen that the most sensitive technique available at present for analysis of environmental samples is AMS which gives the lowest detection limits, three to eight orders of magnitude lower than the radiometric methods (with the exception of ^3^H, where the ^3^He ingrowth mass spectrometry method dominates).

**Table 6:** A comparison of detection limits for frequently analysed long-lived radionuclides in the environment (in Bq)

Method	^3^H	^14^C	^99^Tc	^129^I	^236^U	^237^Np	^240^Pu
RM	10^−3a^	10^−4^	10^−2^	10^−2^	10^−3^	10^−4^	10^−5b^
^3^He MS	10^−3^						
NAA			1	10^−7^		5 × 10^−4^	
ICPMS			10^−5^	10^−3^	5 × 10^−9^	5 × 10^−6^	5 × 10^−6^
TIMS			8 × 10^−6^	10^−8^	10^−10^	10^−9^	0.5 × 10^−6^
RIMS			10^−5^				~ 10^−6^
AMS	10	10^−7^	6 × 10^−6^	10^−10^	10^−10^	10^−10^	0.4 × 10^−6^

^3^He in growth mass spectrometry method

^a^After electrolytic enrichment

^b239,240^Pu

*RM* radiometrics, *ICPMS* inductively coupled plasma mass spectrometry, *TIMS* thermal ionization mass spectrometry, *RIMS* resonance ionization mass spectrometry, *AMS* accelerator mass spectrometry

## Centre for Nuclear and Accelerator Technologies (CENTA)

**Fig. 18:**
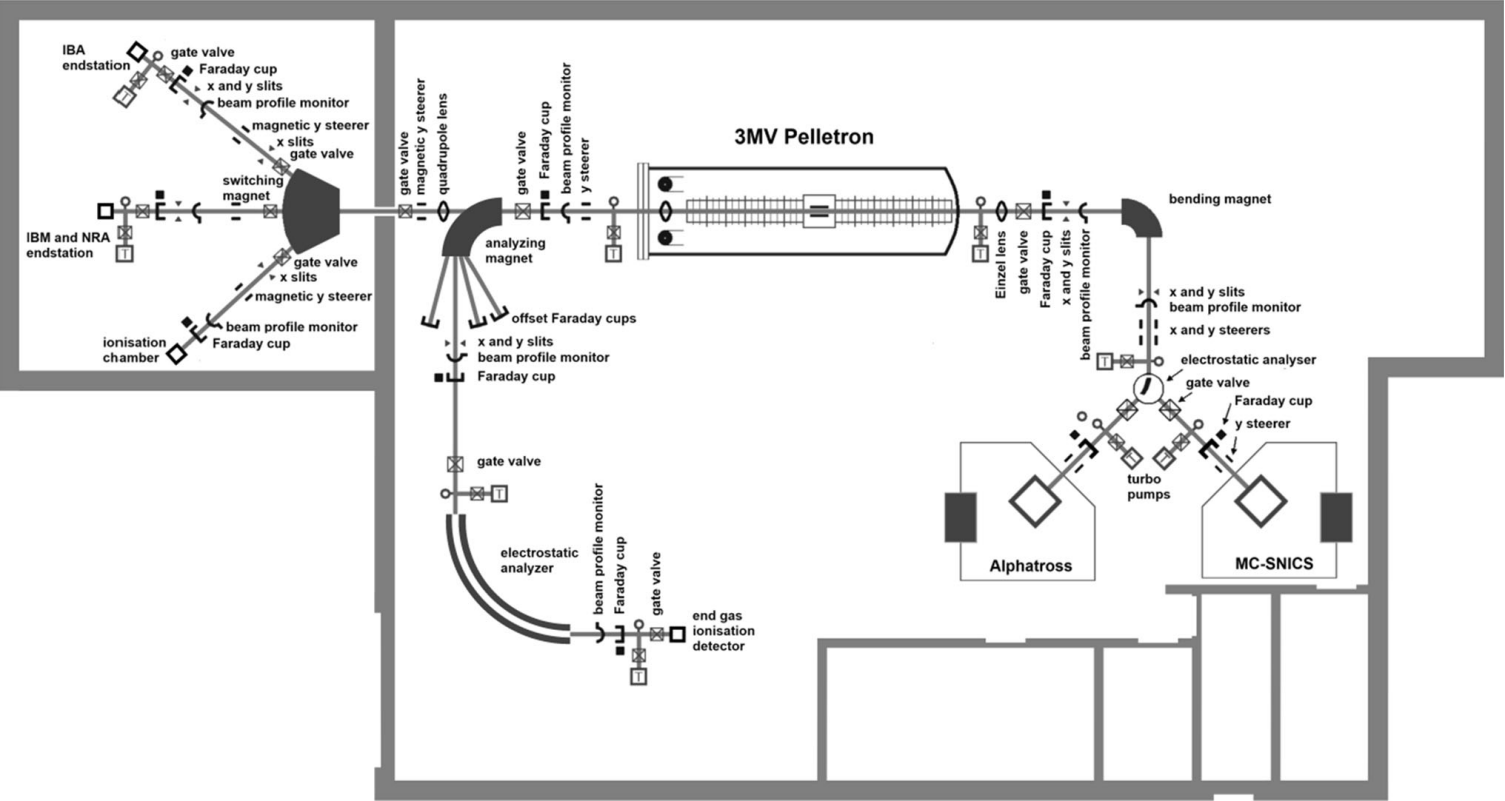
Floor scheme of the CENTA tandem accelerator laboratory with complete AMS line [180]

Recent developments in AMS technologies and their applications in nuclear sciences (physics and chemistry), and in environmental, space, biomedical and material researches have been an inspiration for creation of many new tandem accelerator laboratories. Except for AMS, tandem accelerators have been widely used for the ion beam analysis (IBA) and the nuclear reaction analysis (NRA) of environmental and material samples, as well as for ion beam modification (IBM) of materials. All these new technologies represent the most successful developments in small accelerators and their applications in various branches of science. A Centre for Nuclear and Accelerator Technologies (CENTA) has been established recently (2013) at the Comenius University in Bratislava comprising of a state-of-the art tandem accelerator laboratory designed for: (i) AMS studies of long-lived radionuclides in environmental, space and life sciences; (ii) IBA applications in environmental, life and material research, including cultural heritage studies; (iii) NRA studies with charged particles for new generations of fission reactors, for thermonuclear reactors, and for astrophysics; (iv) and for IBM of materials used for construction of new generation of fission reactors and for thermonuclear reactors, and for research in nanotechnology. This orientation of the laboratory has been driven by general needs to establish in Slovakia a national laboratory devoted to ion beam studies and applications, and to assure for the future wide-range research capabilities for successful participation in international and funding programs. Because of financial constrains it has not been possible yet to install in the CENTA laboratory all equipment which will be necessary for carrying out research in the planned topics. Therefore, the present laboratory design was due to limited financial support restricted to two ion sources (Alphatross for gas, and MC-SNICS for solid targets), the injection system, the 3 MV tandem accelerator, and simple high energy analysers with two ion beam end stations. All available equipment was supplied by the National Electrostatics Corporation (NEC, Middleton, USA). The near future installation will include a fully equipped AMS line with 90° magnet, two electrostatic spectrometers and the end of the line detector (Fig. 18). Later installations will include a nuclear microscope, a raster station for IBM studies of materials, and a station for biomedical research. A dedicated hall to accommodate the tandem accelerator laboratory has been built at the Comenius University campus at Mlynska dolina. The hall design separates the ion beam channels (placed in a bunker covered by soil) from the AMS line, enabling thus work in different radiation environments [179, 180].

The first experiments included optimization of parameters of both ion sources (Alphatross and MC-SNICS) for AMS and IBA studies. Studies of transmission characteristics of accelerated ions with different energy and charge states have also been carried out. The nitrogen gas pressure in the gas stripper for acceleration of ions with various charges and energies has been optimized for different elements. Transmission efficiencies of ^9^Be^2+^ and ^9^Be^3+^ were determined for AMS measurements of ^10^Be. In the first case the transmission efficiency was by more than a factor of 10 greater, therefore the ^9^Be^2+^ beam was used for ^10^Be measurements [181].

### Analysis of ^10^Be by AMS

**Fig. 19:**
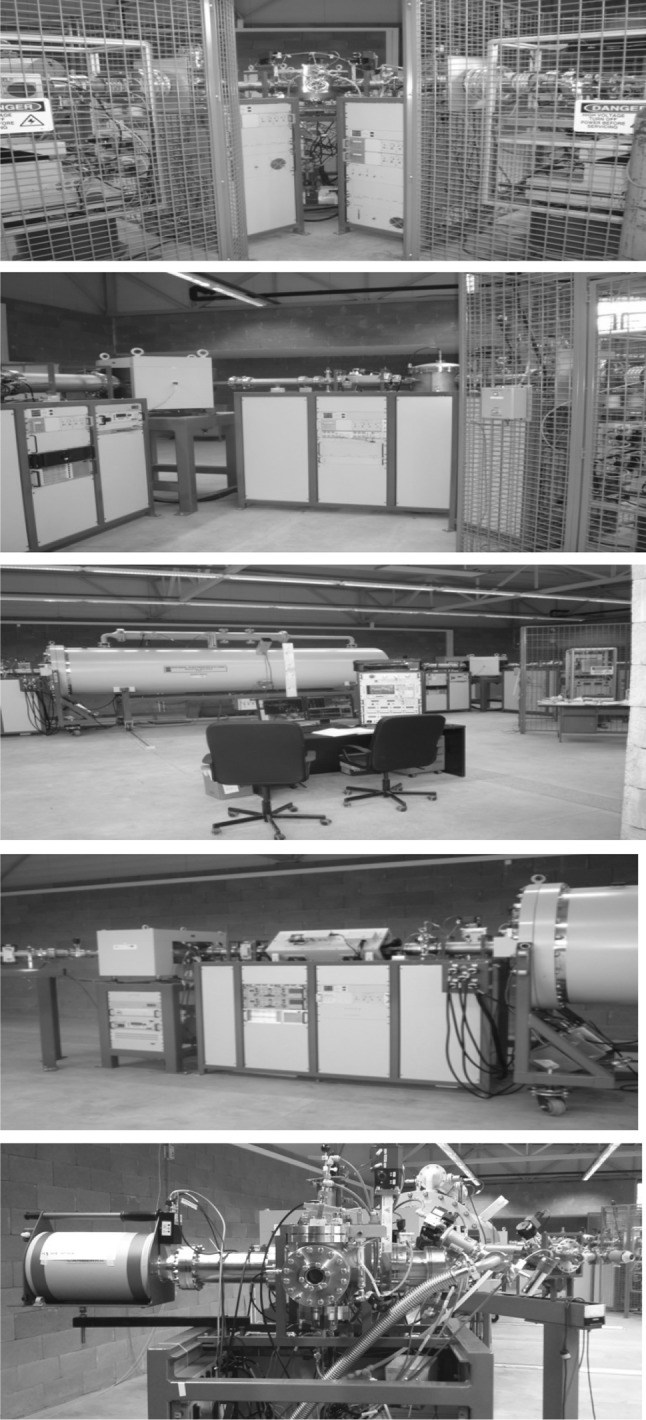
Tandem accelerator laboratory (from the top: Alphatros and SNICS ion sources, injection line, Pelletron, analyzing line with switching magnet, end of the line PIXE/PIGE chamber)

**Fig. 20:**
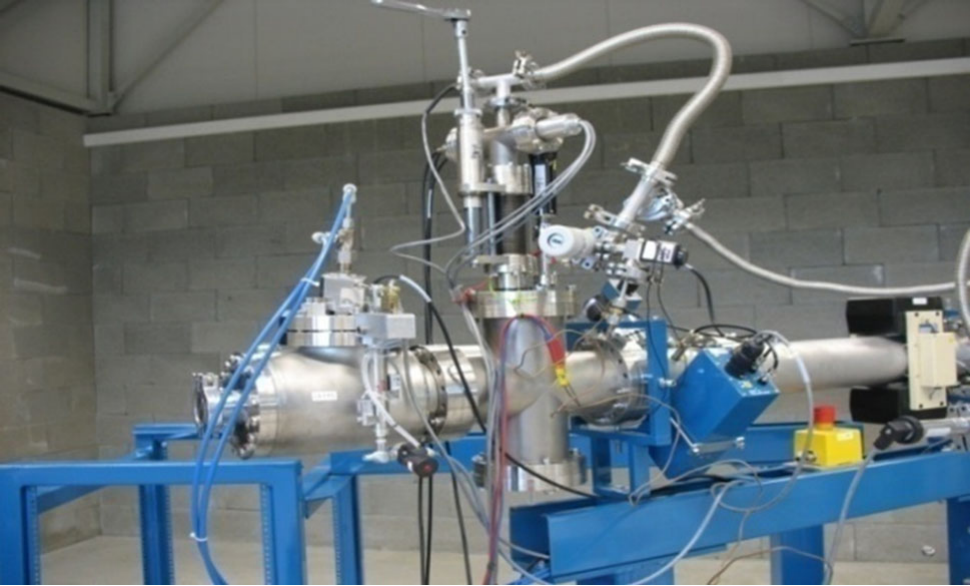
End of the line detectors for ^10^Be analysis

**Fig. 21:**
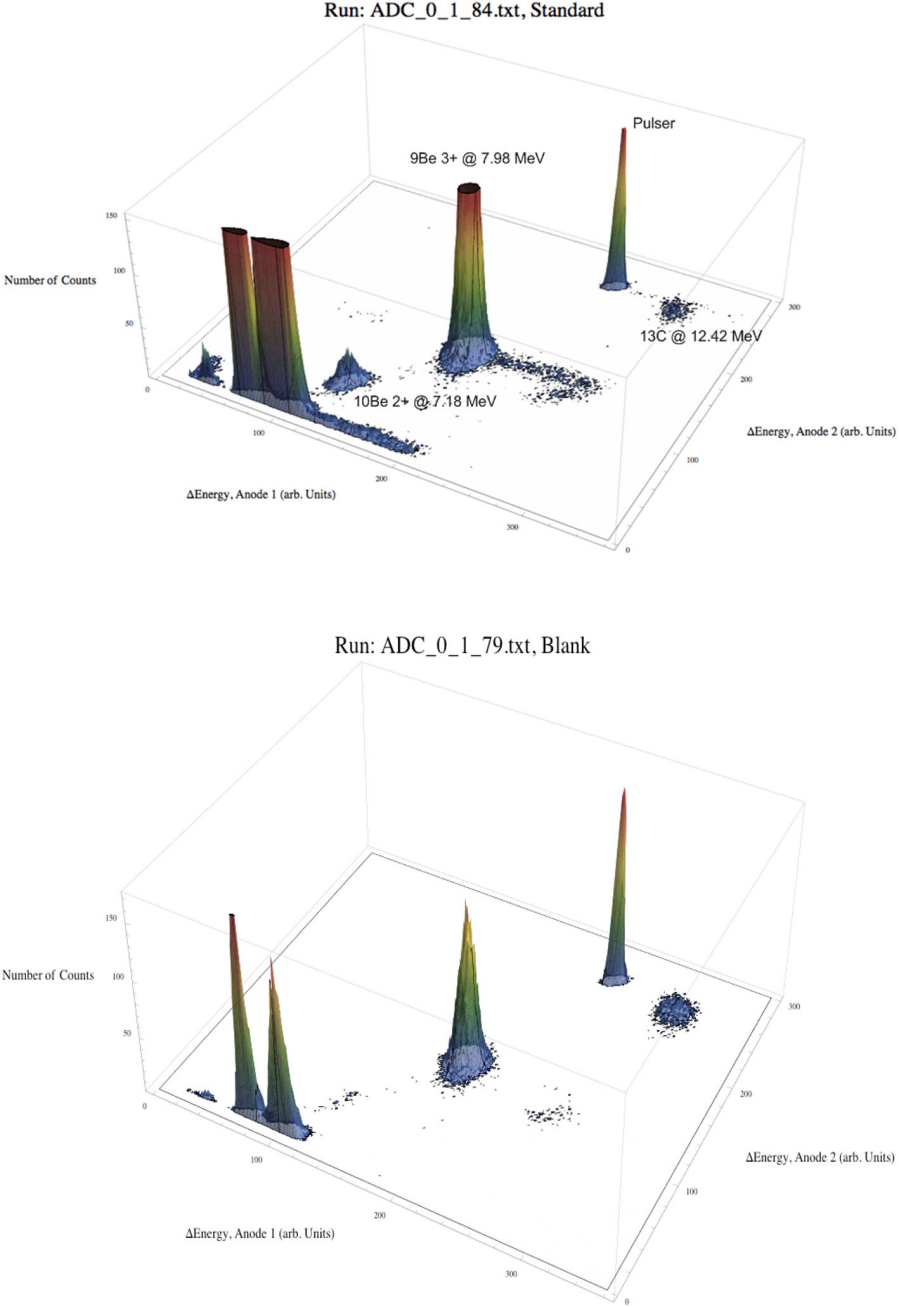
Multidimensional mass spectra of ^10^Be ions (top) and background (bottom) measured in the ionization chamber with silicon nitride stack absorber [181]

As the AMS line at the CENTA laboratory does not yet include a fully capable analysing system, the possibility to measure ^10^Be using only a small switching magnet as the ion analyser was tested. The ^10^Be analysis by AMS is mainly limited by the stable isobar ^10^B, while the requirements for mass separation are the least stringent of all standard radionuclides analysed by AMS. The method for suppression of ^10^B ions, based on a silicon nitride foil stack used as a passive absorber was developed earlier at the VERA laboratory (Fig. 19). The MC-SNICS was used for the production of ^10^BeO^−^ ions, which were mass separated and injected into the 9SDH-2 Pelletron, operating at 3 MV terminal voltage. The ^10^Be^+2^ ions were selected, and ^10^B ions, as well as of most background ions from heavier masses were absorbed in the silicon nitride stack introduced in front of the ionization chamber which was used for the ion detection (Fig. 20). A good separation of ^10^Be^2+^ ions from background (formed mainly by ^9^Be^+2^) has been obtained (Fig. 21). The standard ^10^Be source (S555 developed at ETH Zürich) with the ^10^Be/^9^Be mass ratio of (8.71 ± 0.24) × 10^−11^ was used in these measurements. Using this setup, a detection limit for ^10^Be/^9^Be of 10^−12^ was achieved, which was mainly determined by scattering of ^9^Be^+2^ ions on residual gas inside the switching magnet [181].

### Analysis of uranium impurities in materials

**Fig. 22:**
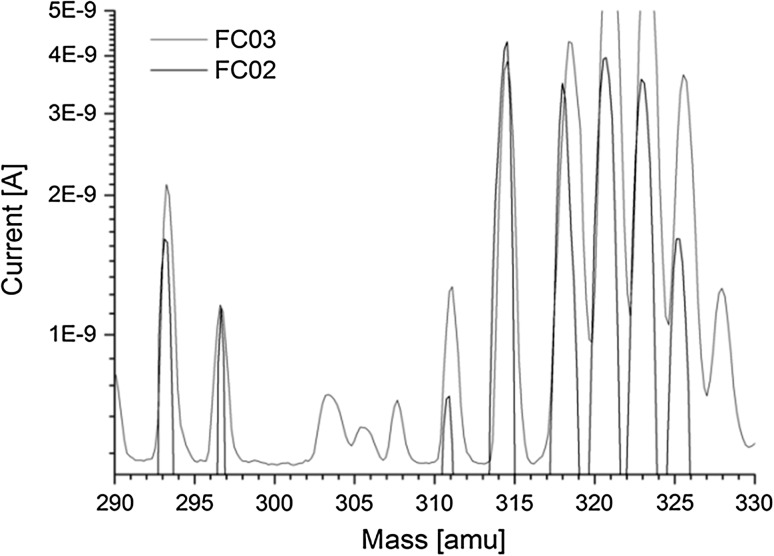
Uranium ions extracted from the copper were either as uranium oxides, or as uranium compounds with copper

**Fig. 23:**
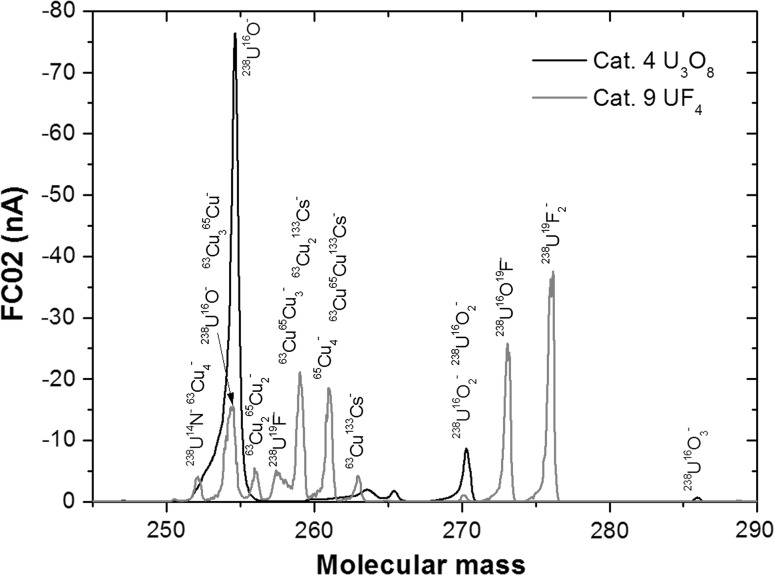
Production of copper clusters after extraction of ions from U_3_O_8_ and UF_4_ targets placed in copper holders

One of the main tasks of the CENTA facility is investigation of U and Th impurities (at levels below 1 nBq g^−1^) in construction materials of detectors designed for underground physics experiments such as double beta-decay of ^82^Se and ^76^Ge (experiments SuperNEMO and LEGEND, respectively), as well as a search for dark matter (experiment EURECA) [182–184]. We have been focusing on radiopurity measurements of copper as this material is usually closest to the detector, and therefore its radiopurity has the dominant impact on the detector background. Copper samples can be directly accommodated as targets in the ion source of the tandem accelerator, therefore no pre-concentration chemistry is required. We did preliminary investigations with analysis of uranium in copper wire targets made of OFHC. Uranium and thorium ions extracted from the copper produce in the ion source negative ions either as uranium/thorium oxides, or as uranium/thorium compounds with copper (Fig. 22) [185]).The ion clusters of ^63^Cu and ^65^Cu (^63^Cu_3_^65^Cu, etc.) with masses of 254 (256), 319 (325) and 374 (388) were observed after the injection magnet, however, the UO and ThO_2_ ions with masses of 254 and 264, respectively, should be expected in the first mass peak as well. This has been expected as U and Th oxides are the most frequently observed compounds of these two elements in the environment, and therefore they will make the most influential background contributions during AMS measurements. A more favourable case should be therefore a formation of negative molecules of UCu^–^ or ThCu^–^ which would fall into the mass windows of 301 and 295, respectively, where they would be free of copper cluster interferences (Fig. 23; the U_3_O_8_ and UF_4_ targets were prepared at the Czech Technical University in Prague [185]). Further investigations are going on with optimization of ion production/acceleration and post-acceleration ion analyses, including analysis of U and Th in enriched ^82^Se which will be used as an isotope source in the SuperNEMO experiment.

### Al_2_O_3_ versus AlN targets for ^26^Al analysis by AMS

**Fig. 24:**
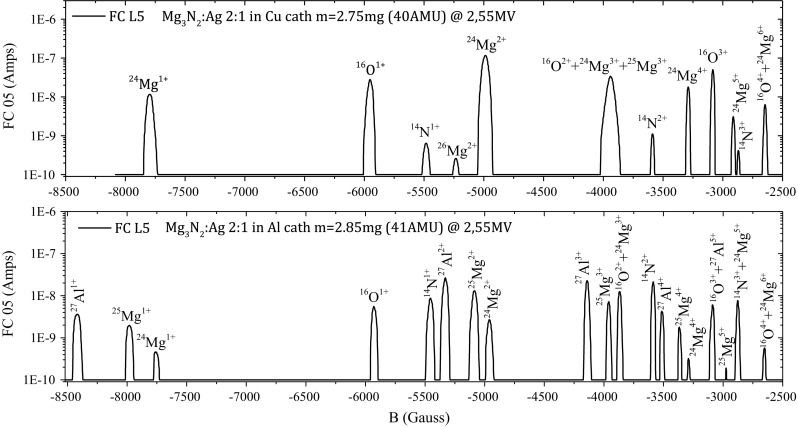
Mass scans of ions from the Mg_3_N_2_ sample after acceleration and dissociation [186]

Al_2_O_3_ targets have been mostly used in ion sources for the production of aluminium negative ions for AMS analysis of low-level concentrations of ^26^Al in environmental, biological and space samples with detection limits down to 0.01 fg. Al_2_O_3_ targets have high-temperature stability, non-toxicity, in-air stability and relatively easy production procedures. It is well known, however, that aluminium does not yield high-intensity negative ion beams, like other elements used in tandem AMS accelerators. An alternative solution could be to use as the target aluminum nitride (AlN) material, which can yield higher Al^–^ currents. On the other hand, the AlN targets are more difficult to synthesize, and they decompose with water in the air to form Al(OH)_3_ and ammonia. Commercially available compounds of Al_2_O_3_ and AlN were mixed with copper, silver and iron high purity powders and sputtered in MC-SNICS ion source for studying ionization yields. Since the production of magnesium and nitrogen negative ions is negligible, a production of MgN^−^ molecules has been questionable. Obtained results indicate that the AlN matrix could be a suitable material for AMS measurements as the production of ^27^Al^−^ is higher by a factor of 2 than from the aluminium oxide matrix, while aluminium sulphate and aluminium fluoride showed a very low sputtering efficiency [186]. Materials such as Al_2_O_3_, AlN, pure aluminium wire and Mg_3_N_2_ were also tested for ^24–26^Mg^14^N^−^ formations on isobaric molecule interference, focusing on searching for interferences at 40 amu (^26^Mg^14^N^−^ creation) as the main isobaric interference for ^26^Al^14^N^−^. In our conditions, none of the isobaric interferences was detected. As can be seen in Fig. 24, there is one small peak that can correspond to the isobaric interference ^26^Mg^2+^ (all samples were ^26^Al blank materials) from injected MgN^−^ (mass 40 amu) ions. The AMS investigations, with the same Al_2_O_3_, AlN and Mg_3_N_2_ matrices, were done in the VERA laboratory using 3 MV terminal voltage. The measurements indicate that the MgN^−^ creation from AlN matrix is unfortunately suppressed only about 3.3 times than the MgO^−^ creation from Al_2_O_3_ matrix. The suppression of MgN^−^ formation from Mg_3_N_2_ matrix was, however, 5 × 10^5^ times lower than AlO^−^ creation from Al_2_O_3_.

### Analysis of ^14^C in annual tree rings by AMS

One of the main goals of the CENTA facility is investigation of ^14^C variations in the atmosphere and biosphere as a continuation of previous research on ^14^C variations in atmospheric CO_2_ of Bratislava (having 50 y tradition and representing the second longest continuing ^14^C record in Europe) [187, 188], as well as around Slovak nuclear power plants in Jaslovské Bohunice and Mochovce [189, 190]. Recently we have been focusing on the development of an integral method of ^14^C variations studies in annually growth tree rings [191]. Such type of sampling enables to reconstruct ^14^C chronology in a given locality, usually for several decades. To reach one-year time resolution, an analytical method based on AMS measurement is necessary, in which a sufficient sample of carbon quantity is only a few milligrams. Another advantage of the AMS based method of ^14^C activity determination is a smaller uncertainty of measurements (reduced by about a factor of two).

#### Preparation of graphite targets for ^14^C analysis by AMS

**Fig. 25:**
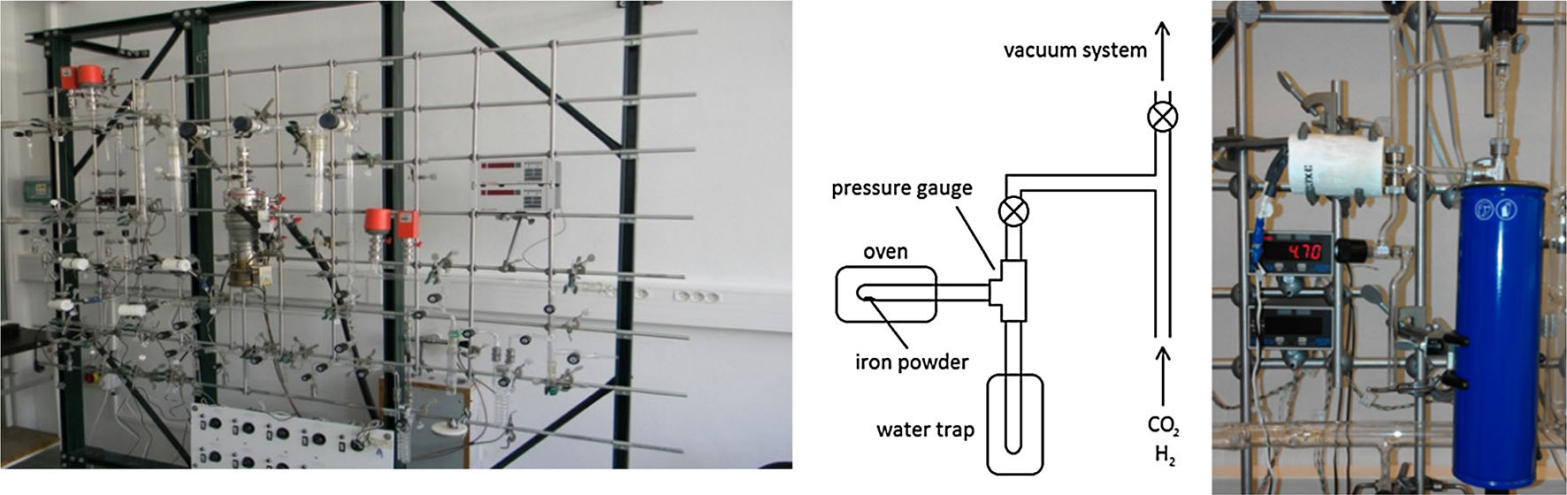
Vacuum-cryogenic apparatus for combustion of tree-ring samples and preparation of graphite samples [191]

Wood samples after extraction from the centre of selected tree rings were treated by the ABA method (1 M HCl 60 °C, 0.1 M NaOH 60 °C, 1 M HCl 60 °C). Dried cellulose separated from the wood was burned in oxygen atmosphere at 900 °C. Cold traps cooled to − 40°C were used for for purification of the CO_2_ (Fig. 25). Small graphitization reactors (0.9 cm^3^) were used for graphite production (Fig. 25) using a modified Bosh reaction [192]. The iron powder was first purified with oxidation at 900 °C before filling the reactors, and then reduced by pure H_2_ at 600 °C. After about 30 min of iron reduction, the reactors were filled with H_2_ and CO_2_ in the ratio 3:1. A part of the reactor was then cooled by the dry ice to − 78 °C. Iron was heated up to 900 °C for the CO production, and after about 30 min, the temperature was lowered to 550 °C for graphite production. Temperature and pressure were monitored during the whole reaction. In addition to the tree-ring samples, chemical blank was prepared by using high purity graphite with the same procedure as the tree-ring samples. Graphite samples produced in the reactors were then pressed into aluminium cathodes and mounted on the MC-SNICS ion source wheel. For calibration purposes, ^14^C cellulose standard IAEA-C3 was combusted, graphitized and mounted together with tree-ring samples and blanks on the wheel. The ^14^C/^12^C ratios were measured in the VERA laboratory using NEC Pelletron accelerator at 2.7 MV terminal voltage [191, 192].

#### Comparison of ^14^C levels in tree rings from Slovakia

**Fig. 26:**
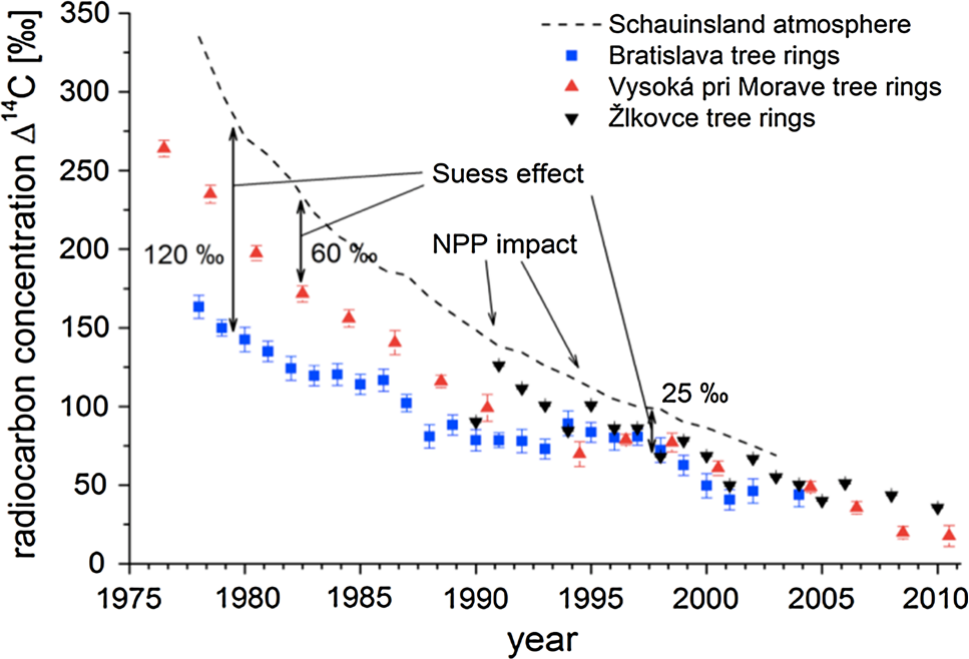
Comparison of ^14^C levels in tree-ring samples from Slovakia with Schauisland atmosphere [192, 197, 198]

Results of ^14^C activity in tree rings sequence lime tree collected at the Žlkovce monitoring station (situated close to the Bohunice NPP, about 60 km north of Bratislava) are compared with Vysoka pri Morave (a village situated 30 km north-west of Bratislava) and Bratislava tree rings (Fig. 26). Due to wood biomass accumulation from May to September, a small decrease in the comparison with maximum activities of atmospheric ^14^CO_2_ (during the summer period) can be expected in tree ring samples. Nevertheless, possible local variations of ^14^C activities caused by microclimatic differences (a time shift in biomass ingrowth) can be reduced for tree rings compared to leaves and other biota samples. The tree-ring method is limited by the age of the tree, but ^14^C data over several decades can be reconstructed from old trees. The ^14^C tree-ring data can provide information on average ^14^C concentrations during spring and early summer (when new wood, light in colour and usually softer, is formed—so called softwoods), and during a late summer and autumn growing season (when new cells formed are smaller, but of a higher density, and have darker thicker walls—so called hardwoods). The tree-rings during the growing season can integrate impacts from both nuclear and fossil CO_2_ sources on the local environment, indicating long-term trends in ^14^C biospheric concentrations, and can be used to assess radiation doses to the public. Radiocarbon concentrations in tree-ring samples from Vysoká pri Morave show an expected exponential decrease during the last 40 years with decay constant of 14.5 ± 1.2 years (*R*^2^ = 0.989), in agreement with similar results obtained at other European ^14^C stations. The Suess effect, represented by a dilution in ^14^C levels by fossil fuel CO_2_ emissions was observed in both tree-ring data sets. The Vysoká pri Morave ^14^C data during 1974–1995 were systematically lower by about 50‰ than the Schauinsland (Germany) clean air reference values due to a regional fossil-fuel impact. However, after 1996 the Vysoká pri Morave ^14^C data were closer to the Schauinsland data [193, 194] due to lower CO_2_ emissions as a result of closing some of the heavy industry technologies in the region. The observed interannual changes in ∆^14^C with considerably larger variances in the Žlkovce than in Vysoká pri Morave ∆^14^C tree-ring data could be caused by small ^14^C releases from the Jaslovské Bohunice NPP. On the other hand, the Bratislava tree-ring ^14^C data have been heavily influenced by fossil fuel emissions (a local Suess effect).

### PIXE beam-line analyses

**Fig. 27:**
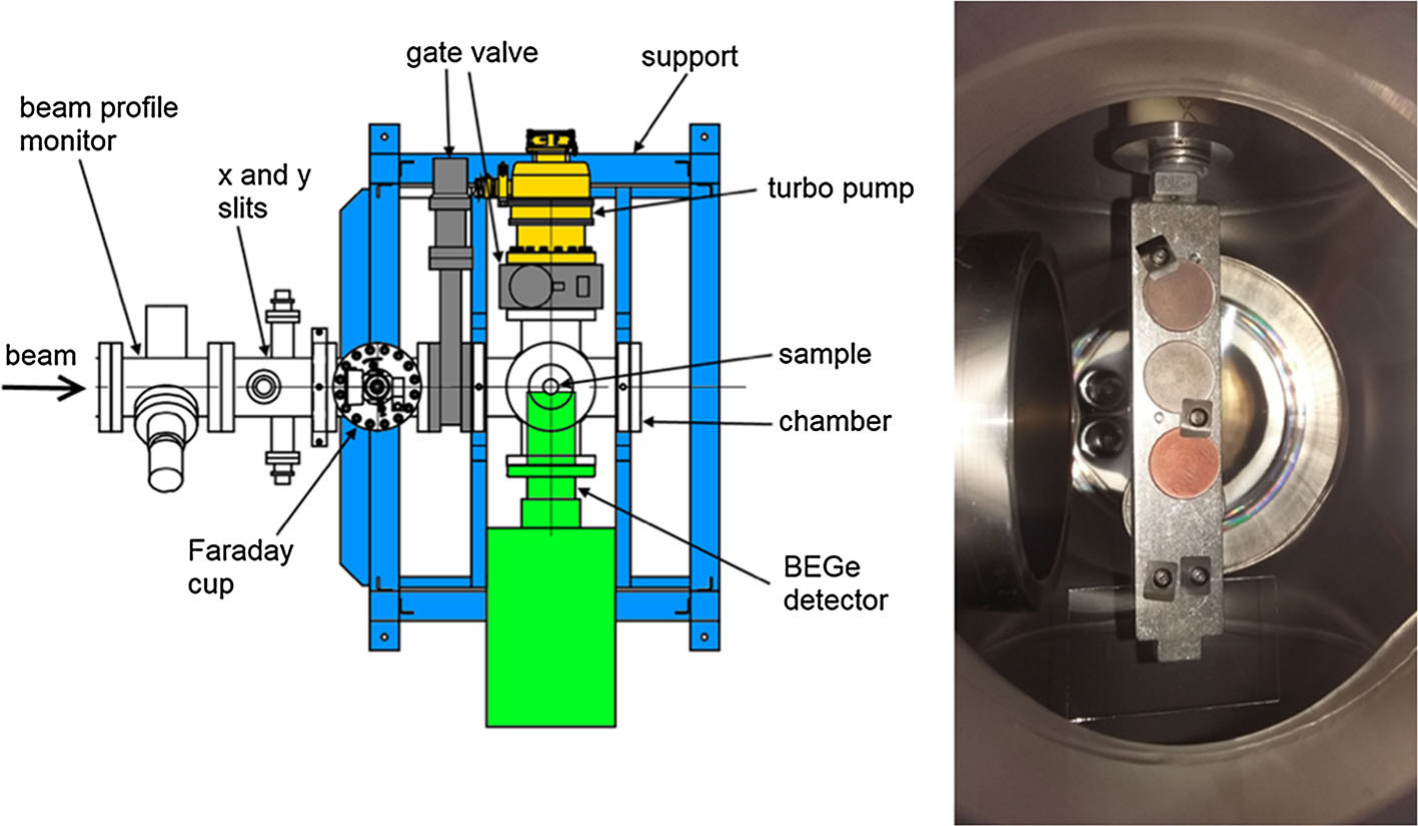
Scheme of the PIXE/PIGE chamber with sample holder and metallic samples. Characteristic X-rays were detected at the 45° angle by the BEGe detector (the endcap with carbon window is visible on the left side of the image) [195]

We shall present first results obtained with the PIXE beam line installed at the Bratislava CENTA tandem accelerator facility. The PIXE reaction chamber is equipped with a vertically movable sample holder for positioning of up to eight samples depending on their dimensions. The holder is capable of rotation around its vertical axis to adjust the angle how the incident beam should hit the sample (Fig. 27). Proton and ^4^He ion beams produced in the Alphatross ion source and accelerated in the 3 MV Pelletron were used in the investigations. Results obtained with the ^4^He ion beam showed better detection limits when compared with protons of the same energy and beam intensity. For detection of produced X-rays, a BEGe detector has been used, covering the energy range from 3 keV to 3 MeV. Analyses of PIXE laboratory standards and old silver coins with ^4^He ions of 3.5 MeV energy showed reproducible results [195].

**Fig. 28:**
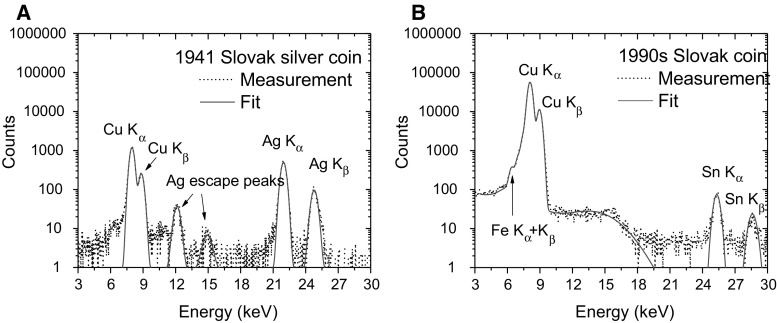
PIXE analysis of two Slovak coins (modified from [195]

PIXE measurements of two Slovak coins using 4.5 MeV helium beam were carried out performed (Fig. 28). A silver coin of nominal value 20 crowns which was issued in 1941 during the Slovak state, and a coin of nominal value 1 crown which was used as a former currency in the Slovak Republic (between 1993 and 2004) were analysed. GUPIX-WIN was used for the composition determination of these coins. The 1941 Slovak silver coin was measured using 50 pA beam intensity for 10 min showed presence of silver (65 ± 5%), and the rest was copper. The copper characteristic X-ray lines (*K*_a_ and *K*_b_) were sufficiently resolved. The second coin measured using higher beam intensity (200 pA) for 20 min showed a dominating copper concentration 85 ± 5%, and the rest was tin (14%) and iron (< 1%) [195].

**Fig. 29:**
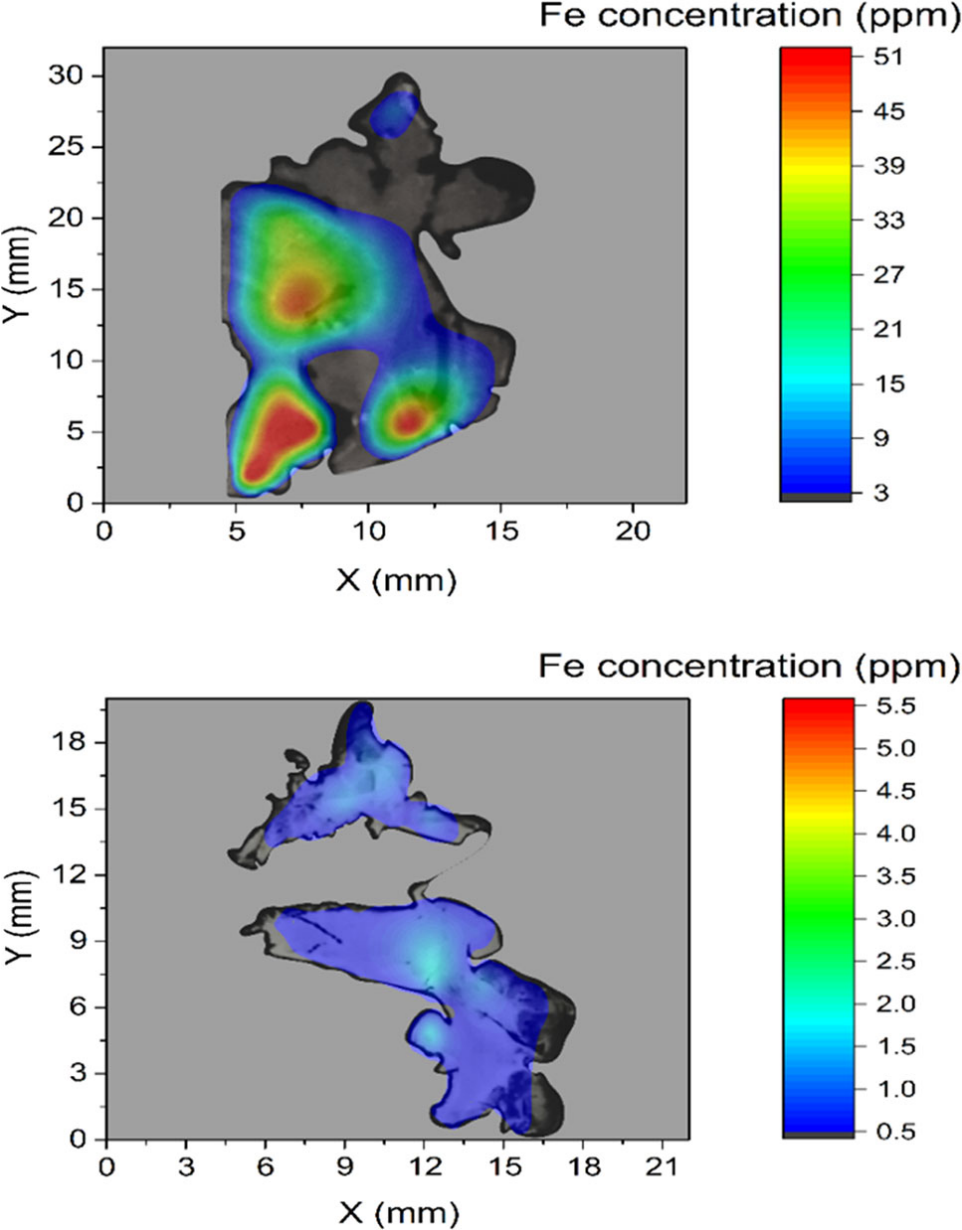
Distribution of Fe in a thin section of rabbit brains after (top) and without (bottom) electromagnetic irradiation

As the second example of PIXE analysis we present measurements of Fe in a section of brain rabbits after electromagnetic irradiation with similar energy and intensity as of mobile phones. There have been long discussions about side effects of frequently used mobile phones for human health, especially possible impacts on children. Figure 29 compares abundance of Fe in the sections of rabbit brains after electromagnetic irradiation and without the irradiation. While the background Fe concentrations were below 2 ppm, the irradiated brain contains aggregations with Fe concentrations up to 55 ppm. A further research is in progress to measure other metals in brains after electromagnetic irradiation including human brains (a paper under preparation).

## Examples of large-scale projects carried out with new radioanalytical technologies

We have only a limited space available to show where new radioanalytical technologies made important breakthroughs in new science. We may just state that in all sciences (e.g. space, environmental, marine, nanotechnologies, biomedical, pharmaceutical, agricultural, etc.), which require radionuclide analyses, the impact of new technologies have been significant as they have enabled to carry out investigations which were not possible before either because of small sample size or the required sensitivity. We shall illustrate the recent developments in the radioanalytical technologies on our recent research activities carried out in the framework of international collaboration, e.g. on investigations of rare decays (SuperNEMO), on atmospheric radioactivity, on isotope groundwater studies and on marine radioactivity studies (SHOTS, ANTARES and FUKUSHIMA projects).

### SuperNEMO and rare nuclear processes and decays

**Fig. 30:**
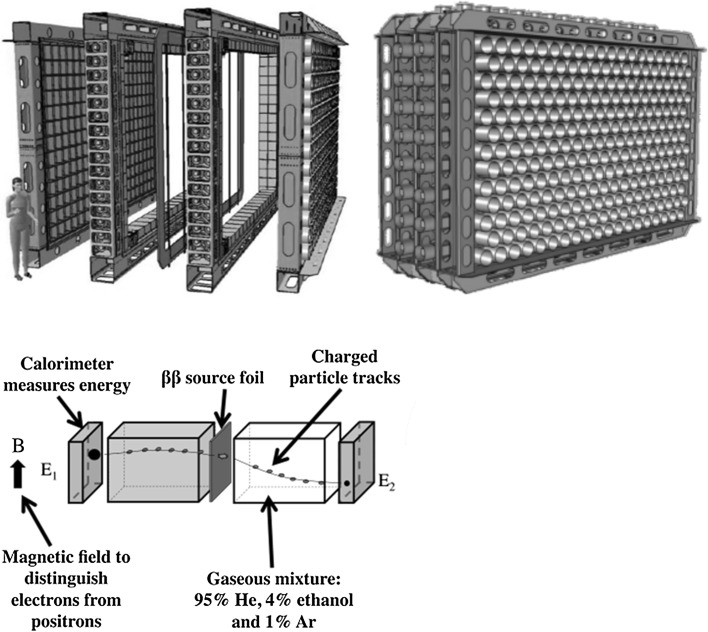
Construction view of the SuperNEMO module with description of its functions (modified from [35])

Background is playing an essential role in underground experiments investigating rare nuclear processes and decays such as neutrinoless double beta-decay experiments (e.g. in SuperNEMO [182] and LEGEND [183]), as well as in search for dark matter (EURECA experiment [184]). As this is a very complex topic, we shall focus here only on the SuperNEMO experiment, which will search for neutrinoless double beta-decay of ^82^Se, and its first modul (called Demonstrator) is presently under construction in the Modane underground laboratory [35, 36, 182]. It is expected that it will be operational at the second half of 2018. The two-neutrino double beta-decay process was observed for several isotopes (e.g. ^48^Ca, ^96^Zr, ^100^Mo, and others, e.g. [196]). The double beta-decay is a second order process (allowed by the Standard Model), and half-lives of the order of 10^20^ years have been reported. However, no positive result has been obtained till now for a neutrinoless double beta-decay which would violate the lepton number conservation, requiring a Majorana neutrino (the identical neutrino with antineutrino), and giving unique information on the neutrino mass hierarchy, representing thus a new physics behind the Standard Model. With more than ten experiments which have been going on in several underground laboratories, only upper half-live limits of the order of 10^24^ years have been reported. The SuperNEMO experiment is further developing an underground physics technology used by its predecessor, the NEMO-3 experiment. It combines tracking and calorimetry techniques for the reconstruction of the final state topology (including timing and kinematics of the double beta-decay transition events), offering thus a powerful tool for background rejection. The SuperNEMO experiment in its full scale will consist of 20 modules (8 m high, 7 m long and 6 m wide) having the source foil situated in the centre of the module, tracking chambers at each side and finally calorimeters at each side for energy and time of flight measurements (Fig. 30). The selenium powder (^82^Se enriched source) is distributed on the foil with surface density of 53 mg cm^−2^. After the enrichment, it has been chemically purified using extra pure materials at super clean conditions. All the materials entering the source production have been carefully selected and prepared to have ultra-low radionuclide contamination levels.

The background constrains of the SuperNEMO detector are determined by external and internal radiation sources. The external ones are mainly due to cosmic rays (muons, neutrons and gamma-rays), neutrons from the underground laboratory walls, gamma-rays from the construction parts of the detector, and radon contamination of the laboratory air, which can also infiltrate inside the detector. The background from internal sources will be mostly composed of: (i) Double beta-decay events, which may imitate neutrinoless beta-decay; (ii) radioactive contamination from the internal construction parts, mainly in the tracker; (iii) radon inside the tracker. While the first constrain can only diminish by high quality spatial, timing and energy resolution capabilities of the SuperNEMO detection system, the last two constrains require using as clean as possible radioactivity free construction materials. Radon (^222^Rn) and its decay products (^214^Bi and ^214^Po), as well as decay product of thoron, ^220^Rn (^208^Tl) have been identified as the most dangerous contaminants for the SuperNEMO detector. The most sensitive parts of the SuperNEMO detector from the point of view of radioactive contamination are: (i) isotope source (^82^Se); (ii) supporting foil for the isotope source, and (iii) tracker (mainly the components which are inside the tracker, i.e. anode wires, cathodes, front/end insulators). The radon and thoron inside the tracker will decay to ^214^Bi and ^208^Tl, respectively, and their decay products will deposit on source/foil surfaces and on anode wires. They can also infiltrate in other parts of the detector, and their decay products due to emitted beta-electrons with high energy (up to 3.3 MeV and 4.9 MeV for ^214^Bi and ^208^Tl, respectively) and gamma-rays (up to 2.2 and 2.6 MeV for ^214^Bi and ^208^Tl, respectively) may imitate double beta-decays. The tracker amelioration mainly relies on the reduction of the radon presence inside the tracker volume. To achieve this reduction, it is necessary to perform an exhaustive control of the radon-tightness and radon-emanation of the different materials conforming the tracker. After the installation of the tracker modules, the remaining radon activity inside the tracker should < 0.15 mBq m^−3^, requiring a continuous purification of the counting gas in the tracking chambers from radon. Radon adsorption materials have also been tested to improve the purification systems, as well as to build at the LSM a radon-free air factory that will flush the air around the SuperNEMO detector. Following the experience from the NEMO-3 experiment, the radiopurity limits defined for the SuperNEMO experiment are based on the goal to reach for the neutrinoless double beta-decay the half– life of 1 × 10^26^ y. The stringent limits are set for ^222^Rn present in the tracker (< 0.15 mBq m^−3^), for the radioactive contamination of the internal parts of the detector (the isotope source, supporting foil, wires and walls of the tracker) they are < 2 nBq/g and < 10 nBq/g for ^208^Tl and ^214^Bi, respectively. Although ^40^K also emits high-energy gamma-rays (1.46 MeV), and it is frequently present mainly in glass of photomultipliers, it is usually outside of the sensitive volume of the tracker, and therefore radiopurity limits for ^40^K are not so strict (< 100 mBq kg^−1^). When compared with the NEMO-3 detector, the radio-contamination limits for the SuperNEMO are lower by about a factor of 30, 50 and 30 for ^222^Rn, ^208^Tl and ^214^Bi contamination, respectively.

Low-level Ge gamma-spectrometry facilities were developed underground in LSM (Modane, France) and in Boulby (Cleveland, UK), and surface ones in Bordeaux and Bratislava to check radiopurity of construction materials. Table 7 lists as an example a few results of recently carried radiopurity measurements of construction materials.

**Table 7:** Recent radiopurity measurements of construction materials

Radionuclide	Activity (nBq/g)								
	Mylar foil^a^	Plastic scintillator^b^	Ultra-clean copper^c^	Clean copper	M-copper^d^	Electrolytic copper^f^	Signal cables^b^	Tracker wires^b^	PMT glass^b^
^232^Th	0.9 ± 0.2	< 100	0.034 ± 0.008	60 ± 20^d^ < 19^e^	80 ± 30	1600 ± 200	< 1000	850 ± 180	65,000 ± 7000
^238^U	1.0 ± 0.3	< 300	0.13 ± 0.04	1.0 ± 0.5^d^ < 16^e^	2.1 ± 1.1	< 1600	< 1000	410 ± 90	4000 ± 500
^40^K		2200 ± 1000					25,000 ± 4000	5600 ± 2700	150,000 ± 11000

^a^BiPo-3 [197]

^b^HPGe [36]

^c^ICPMS [198]

^d^AMS [199]

^e^HPGe [56]

^f^NAA [200]

The SuperNEMO experiment is the new generation of tracking-calorimetry neutrinoless double beta-decay experiments. No background event is expected in the neutrinoless double beta-decay region in 2.5 years of Demonstrator operation using 7 kg of ^82^Se. The half-life sensitivity is expected to be *T*_ββ_^0ν^ > 6.5 × 10^24^ years, corresponding to an effective neutrino mass sensitivity of |0.2–0.4 eV| (90% C.L.). The full SuperNEMO experiment comprising of 20 modules with 100 kg of ^82^Se source should reach a neutrino mass sensitivity of |0.04–0.1 eV| (90% C.L.), and a half-life limit of (1 × 10^26^ years).

New generations of underground experiments will require, however, further developments of ultra-sensitive radioanalytical technologies. The big problem of the gamma-ray spectrometry for ultra-low-level radioactivity screening is the fact that the same radionuclides, which should be analysed in construction materials are also found in the detector background. Therefore, alternative methods for analysis of primordial radionuclides (mainly ^238^U and ^232^Th and their decay products) in construction materials have been suggested. The revolutionary approach in the radiometric sector has been the BiPo coincidence spectrometer, where already a third generation of this ultra-sensitive detector system has been developed [197]. The detector can measure ultra-low levels of alpha- and beta-emitters on large surface materials (e.g. supporting foils, ^82^Se isotope source on the foil) used in the SuperNEMO experiment. To gain in sensitivity, the principle is to detect the delayed beta-alpha coincidences of the ^214^Bi–^214^Po cascades (^214^Bi is the main contributor to the SuperNEMO detector background). The high-energy gamma-emitter ^208^Tl is qualified in the BiPo detector through its mother product, the ^212^Bi. The detector consists of 2 face-to-face planar calorimeters made of pure aluminized polystyrene scintillators coupled to 5′′ low radioactivity PMTs to detect the beta- and alpha-particles, and measure the time delay to distinguish the two isotopes. The total surface area of the detector is 3.6 m^2^. The BiPo-3 detector is operating since 2013 in the Canfranc underground laboratory in Spain.

The present state-of-the-art detection limits of the ultrasensitive detection methods are compared in Table 8. It can be seen that the leaders are AMS, ICPMS and BiPo-3. It would be advantageous if no pre-concentration treatment of samples would be carried out, as this process could add radioactive contamination from chemicals used during sample processing. From this point of view the AMS could be the preferably technique as samples such as copper, steel, etc. can be directly used as targets in ion sources, while ICPMS analyses would always require a pre-concentration chemistry. Similarly, NAA could be used without pre-irradiation chemistry as well, having also advantages in post-irradiation chemistry, which could improve detection limits.

**Table 8:** A comparison of detection limits for ^238^U and ^232^Th

Radionuclide	Detection limits (µBq)					
	Alpha-spectrometry^a,h^	Underground gamma-spectrometry^i^	BiPo-3^d,i^	ICPMS^h^	AMS^f,i^	NAA^g,i^
^232^Th	100	5000^b^ 2400^c^	1.4	0.003^e^	0.0002	0.08
^238^U	100	4000^b^ 2000^c^	1.6	0.010^e^	0.0001	0.2

^a^Estimated from [111]

^b^Estimated from [201]

^c^Estimated from [202]

^d^Estimated from [197]

^e^Estimated from [198]

^f^Estimated from [199]

^g^Estimated from [200]

^h^With pre-concentration chemistry

^i^Without pre-concentration chemistry

### Radioanalytical impacts on environmental studies

New ultra-sensitive radioanalytical technologies made important breakthroughs in environmental sciences, however, we can present in this review only a few examples of successful studies tracing radionuclides in the atmosphere, groundwater and in the marine environment.

#### Atmospheric radioactivity

**Fig. 31:**
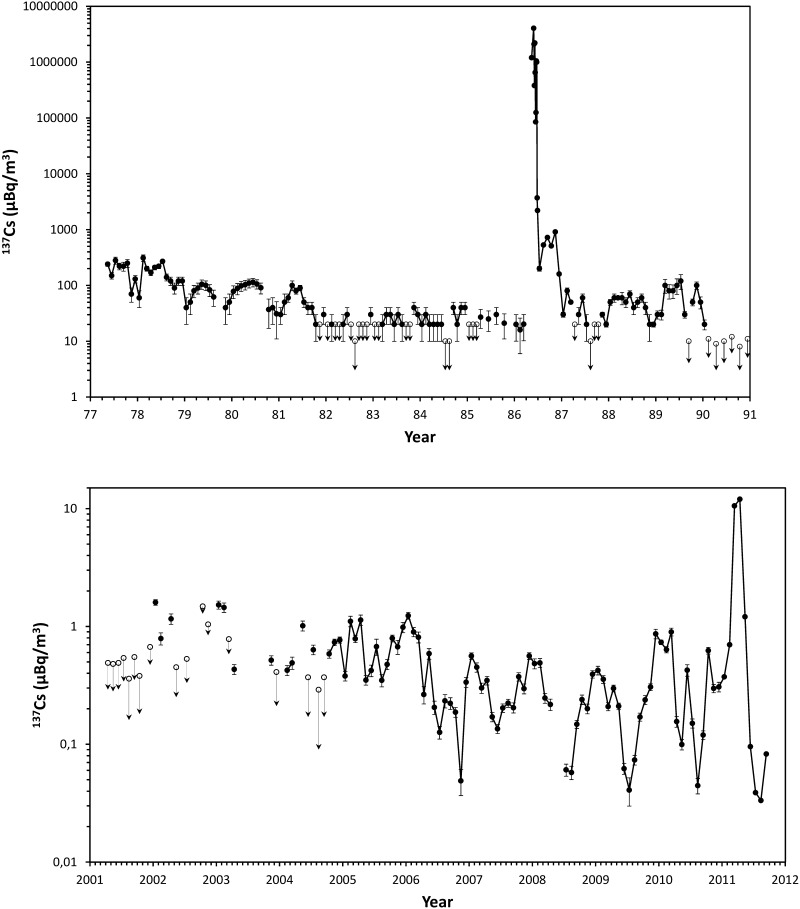
^137^Cs activity concentrations in atmospheric aerosols in the Bratislava air (Slovakia, Central Europe) (modified from [207]

We shall focus on investigations of ^137^Cs from global fallout, Chernobyl and Fukushima accidents as ^137^Cs has been considered to be the most important radionuclide for the long-term radiological impact after nuclear accidents mainly because of large releases, its relatively long physical half-life and its bioavailability. Thus because of its high abundance and accumulation in human tissue, it has been important for delivering radiation doses to the public [203, 204]. The ^137^Cs in the terrestrial environment is present in atmospheric aerosols, soil and vegetation, and it has been used worldwide as a tracer of environmental processes, specifically for studying transport process in atmospheric, aquatic and terrestrial ecosystems. The ^137^Cs after its release to the atmosphere is rapidly associated with aerosol particles, which represent a major reservoir of pollutants in the atmosphere. A new generation of high-sensitive Ge spectrometers, operating under optimum shielding conditions, have been able to carry out ^137^Cs activity concentration measurements down to 0.1 μBq m^−3^, decreasing thus atmospheric aerosol sampling time to several hours. The ^137^Cs released from all sources (mainly global fallout and the Chernobyl accident) has primarily been deposited on the Earth’s surface by dry and wet deposition. Before the Chernobyl accident, temporal variations of ^137^Cs in the surface air were strongly related to global fallout, which was stratospherically controlled [205], i.e. having maxima in late spring and early summer months (Fig. 31) due to transport of stratospheric air to the troposphere. Concentrations of ^137^Cs in the atmosphere had a decreasing trend after the moratorium on testing nuclear weapons in the atmosphere signed in 1963.

The situation has changed in 1986 when due to the Chernobyl accident a high peak appeared in ^137^Cs activity concentration in the surface air (Fig. 31) [206]. The Chernobyl accident released about 85 PBq of ^137^Cs to the atmosphere [4], representing thus the largest single release of ^137^Cs which occurred till now. The maximum ^137^Cs levels observed in the beginning of May 1986 in the Bratislava air were 4 Bq m^−3^, about 200 000 higher than the pre-Chernobyl value (Fig. 31). A few years after the Chernobyl accident the summer maxima, characteristic for the transport of ^137^Cs from the stratosphere to the troposphere were still visible, later, during the 1990 s the main source of atmospheric ^137^Cs became the soil resuspension and its transport in the ground-level air [192].

The Fukushima accident caused in Europe only a negligible increase in ^137^Cs activity concentration in the ground level air. The pre-Fukushima ^137^Cs levels measured during the 2010–2011 in Bratislava ranged from 0.04 to 1.0 μBq m^−3^ (Fig. 31) [207]. Radionuclide activity concentrations observed in the Bratislava air showed three concentration maxima which were associated with different air masses present in the central Europe. When compared with the Chernobyl data (4 Bq m^−3^) we see that they were at least by four orders of magnitude lower. This was mainly due to a long transport of air masses from Japan to central Europe, as well as by about a factor of 5 lower release rates of ^137^Cs to the atmosphere during the Fukushima accident [4]. Therefore, estimated radiation doses to the public of Europe were negligible, around six orders of magnitude lower than the accepted limit of 1 mSv year^−1^.

#### Radiocarbon in groundwater of the Danube River Basin

**Fig. 32:**
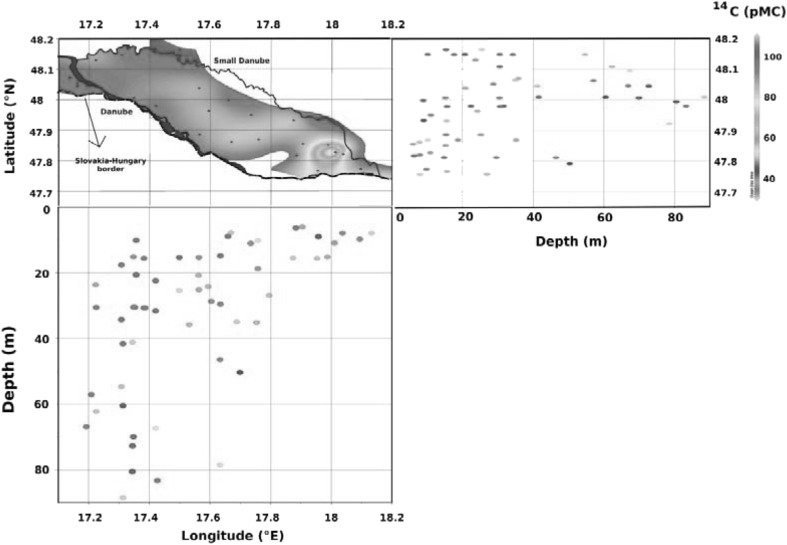
Spatial and vertical distribution of ^14^C with latitude and longitude in groundwater of Žitný Island (modified from [208]

Isotope tracers (^14^C, ^13^C and ^18^O) have been applied for better understanding of the groundwater origin at the Žitný Island situated in the south-western Slovakia between the Danube and Small Danube rivers. The spatial distribution of ^14^C in shallow groundwater (in the form of dissolved inorganic carbon) of the Žitný Island (down to 20 m depth) is presented in Fig. 32 [208]. The observed values are mostly > 80 pMC (percent of modern carbon), except for two wells (depths of 10.9 and 15.2 m) on the east side of the island where the ^14^C values of 31.5 and 69.9 pMC, respectively, were obtained, indicating that in this region we are dealing with a groundwater reservoir which has been outside of the direct influence of the Danube River. The core of the ^14^C profiles represents, however, modern groundwater as the majority of groundwater has ^14^C content above 80 pMC. The groundwater levels at Žitný Island have been depending therefore on water levels (and flow volumes) in the Danube River. This has been well manifested in the central and western part of the island where the thickness of Quaternary sediment is tens (even hundreds) of meters. However, in the eastern part of the island (where groundwater with lower ^14^C content was observed), Neogene clays were found a few meters below the surface, which prevent a direct infiltration of groundwater of Danube origin to deeper layers. This would indicate the existence of a confined aquifer formed below the layer of Neogene clay sediments. This is not the case in the central and western parts of the island, where the Quaternary sediments are much thicker, and where mainly the Danube River system is directly influencing the groundwater regime of the Žitný Island, even at depths down to 90 m. The obtained results on spatial and vertical variability of ^14^C, suggest isotopic heterogeneity in the groundwater of Žitný Island. This has been a first attempt in the isotope hydrology to construct isotope maps and to study spatial and vertical distribution of isotopes in groundwater. We hope that this new research approach will improve the capability and efficiency of using isotopic tools for deeper evaluation, more rigorous assessment, and more efficient management of water resources in the future as fresh water becomes a strategic source of living in the future.

### Global marine radioactivity studies

Circulation of water masses in the world ocean play a crucial role in the protection of the marine environment against contamination from land based sources, as well as for climate change studies, in which oceans are dominant players. A global thermohaline circulation (conveyor belt) has been proposed as a global ocean circulation model connecting ocean basins with surface (warm) and bottom (cold) waters. This represents a transport of warm surface waters from the North Pacific through the Indonesian throughflow into the Indian Ocean and then via the Agulhas Current system to the Atlantic Ocean. Ocean has also been a significant sink of global fallout radionuclides (e.g. ^3^H, ^14^C, ^90^Sr, ^137^Cs, Pu isotopes) which have been injected onto the ocean surface from the atmosphere mainly in the 1960 s, after large-scale US and former USSR atmospheric nuclear weapons tests carried out in the atmosphere. The major deposition of global fallout radionuclides occurred in the northern hemisphere, where about two third of cumulative deposit of global fallout radionuclides was found. Mapping of this deposition revealed that the major injection on the ocean surface occurred at mid- latitudes of the western North Pacific. It has been recognised in the past that global fallout ^137^Cs is powerful tracer for tracing exchange of seawater between the ocean basins because due to global fallout higher deposition regions are found in the western part of mid latitudes in the North Pacific [209]. Concentrations of global fallout radionuclides in seawater have been measured since the 1950 s with the aim to assess radiological impact from nuclear weapons tests on the marine environment and humans. Later, these radionuclides have been used as transient tracers to investigate a movement of water masses in the World Ocean on time scales from years to hundreds of years, depending on their half- lives and residence times in the ocean. A lot of marine radionuclide data have been collected during the past five decades, which have been recently stored in marine radionuclide databases [210–212]. Although the radionuclide data are temporally and spatially heterogeneous, due to their snap shot injection onto the ocean surface, they provide unique opportunities to trace water masses and to study biogeochemical processes in the water column, what has not been possible before because of absence of high-sensitive radioanalytical methods. We shall illustrate the impact of radioanalytical technologies on our recent research activities carried out in the framework of international collaborations in the World Ocean.

#### Southern hemisphere ocean tracer studies (BEAGLE2003 expedition)

The Southern Hemisphere Oceans Tracer Studies (SHOTS) project was carried out during 2002–2010 [107–109, 213] with the aim to collect radionuclide water profile data for better understanding of circulation processes in the south oceans. Seawater profile samples were collected during the round the globe BEAGLE2003 (Blue Ocean Global Expedition) and analysed for ^3^H, ^90^Sr, ^137^Cs and Pu isotopes, as well as for other tracers (e.g. nutrients). The project was carried out by the international collaboration comprising IAEA (Environment Laboratories, Monaco), Japan (Meteorological Research Institute, Tsukuba; Sophia University, Tokyo; University of Kanazawa) and Slovakia (Comenius University of Bratislava). Several radioanalytical laboratories participated in the project as well. The results of this study were published as a special issue of the journal Progress in Oceanography [107–109, 213]. The SHOTS study represents a new approach in isotope oceanography, important for better understanding of oceanographic processes and climate change manifestations in the marine environment. As an example of low-level gamma-spectrometry applications in marine sciences we shall present high density ^137^Cs profiles in the water column of the world ocean. Analyses of 10-20 L seawater samples were carried out using high-efficiency Ge detectors operating in underground laboratories with or without anticosmic shielding [1–3, 39, 40, 60, 168]. These are the first high density ^137^Cs profiles obtained till now for the main oceanic basins. It has been possible to get such profiles thanks to sampling of seawater profiles with Rosette systems, what was not possible before, as large volume samplers (> 100 L) were required. The sampling of water column at large volumes resulted in long sampling times, which had strong financial limitations as a ship time became too expensive.

**Fig. 33:**
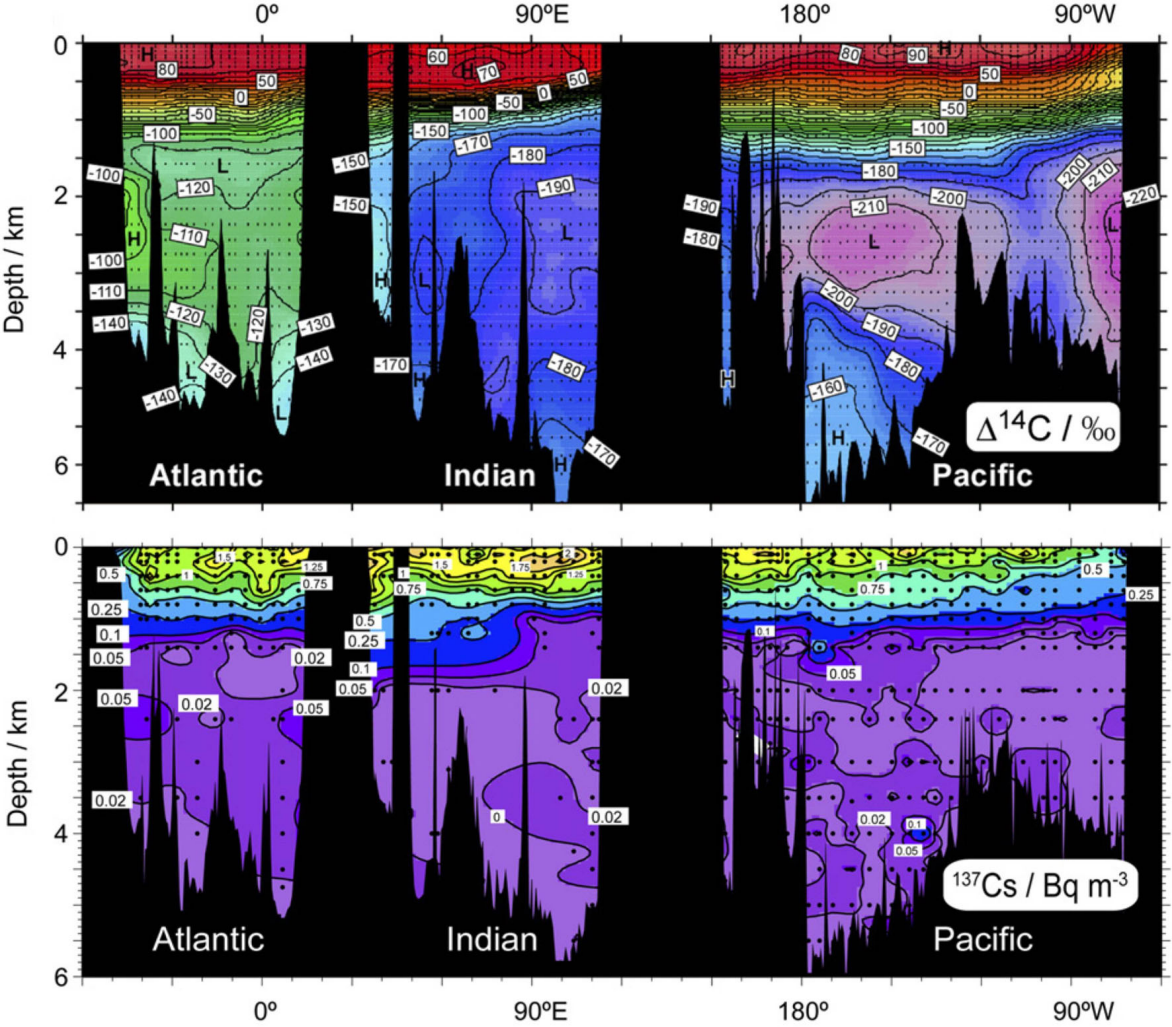
^14^C and ^137^Cs profiles in the world ocean waters across Pacific (30°S), Indian (20°S) and Atlantic (30°S) Oceans. The observed ^137^Cs levels indicate transport of water masses from the North Pacific to the South Pacific (to the Tasman Sea), via Indonesian Seas to the Indian Ocean, and then via Agulhas current to the South Atlantic Ocean (modified from [214])

The ^137^Cs transect along the 20°S latitude in the Atlantic, Indian and Pacific Oceans is shown in Fig. 33 (combined in [214] using SHOTS/BEAGLE data [107–109, 213]). The main feature is a transport of global fallout ^137^Cs from the north-west Pacific Ocean to the South via the Equator, and its accumulation in the Tasman Sea. The ^137^Cs labelled waters are then transported via the Indonesian Seas to the Indian Ocean, where they are again accumulating in the Subtropical gyre operating at 20° and 40°S. Finally, the ^137^Cs is transported by the Agulhas current system to the South Atlantic Ocean, where it became a part of the south Atlantic circulation. Figure 33 also shows ^14^C profiles in the World Ocean obtained in the framework of the SHOTS project [213, 214]. We can see that the ^3^H profiles obtained 10 years earlier for the Indian Ocean have similar features, e.g. the existence of ^14^C minima in each basin. The both ^14^C and ^137^Cs sections provide valuable information on differences in spreading of water masses between the main oceanic basins, and from the surface into the interior of the World Ocean.

#### Water masses in the south Indian Ocean (ANTARES expedition)

**Fig. 34:**
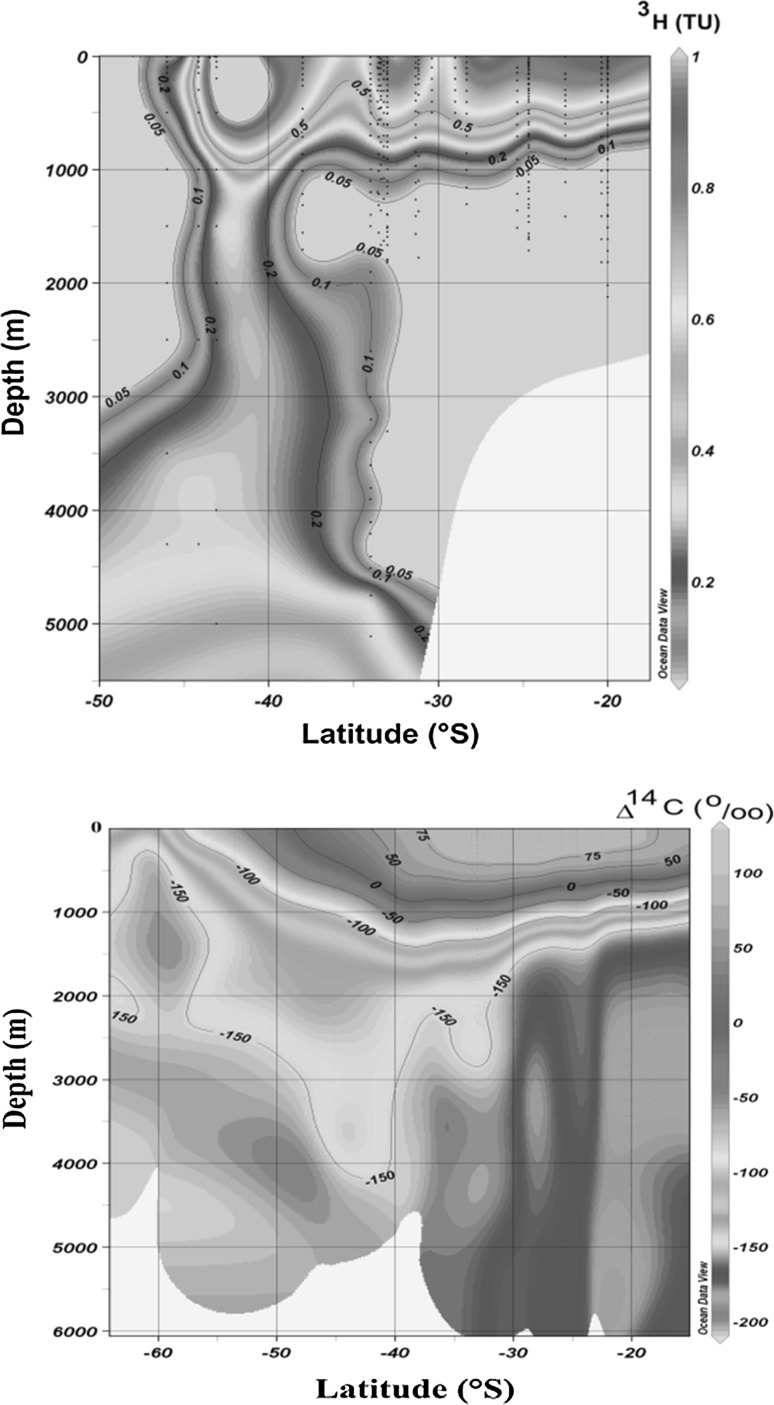
Spatial distribution of ^3^H and ^14^C in waters of the South Indian Ocean (ANTARES data were combined with nearest WOCE stations). Downward tritium and radiocarbon transport around 40°S (at 60°E) can be seen in these profiles (modified from [32]

Ocean observations and water sampling was carried out as a part of the ANTArctic RESearch (ANTARES) IV cruise offshore of the northwest of Kerguelen Islands and east of Crozet Islands between 32°–48°S and 51°–70°E [32]. The ^14^C profile shows a downward transport of bomb produced ^14^C to the depth of about 2000 m. The spatial distribution of ^3^H in the south Indian Ocean (Fig. 34, modified from [32]) suggests that the highest levels were observed in the main stream of the Subtropical gyre, a clear influence of the gyre on the downstream radionuclide transport from its western boundary associated with the Agulhas Retroflection. The ^3^H water profiles across the basin, combined with WOCE data south of 34°S (decay corrected to 1999), show higher surface and subsurface ^3^H levels at 20°S and 40°S latitude belts. This indicates an accumulation of ^3^H within the Subtropical gyre on a time scale of several decades. The gyre acts as a reservoir, maintaining higher radionuclide concentrations in the region. The ^14^C water profile data clearly shows a penetration of bomb ^14^C around 40°S (transect at 60°E) down to almost 5000 m (similarly as we could see it in the case of tritium), what documents that the south Indian Ocean is important for sink of anthropogenic carbon. The south Indian Ocean have been acting on a time scale of several decades as a final reservoir of contaminants transported from the northern Indian and Pacific Oceans, which will cause that all garbage present in the world ocean will be finally stored in the Subtropical gyre of the Indian Ocean. The observed distribution of isotopic tracers in the Crozet Basin reflects the complex dynamics and advection of different water masses, which makes the basin one of the most interesting oceanographic places in the world ocean. The world ocean generally control the climate on the Earth, but the Indian Ocean in this respect is playing there a crucial role.

#### Fukushima impact on the Pacific Ocean waters

**Fig. 35:**
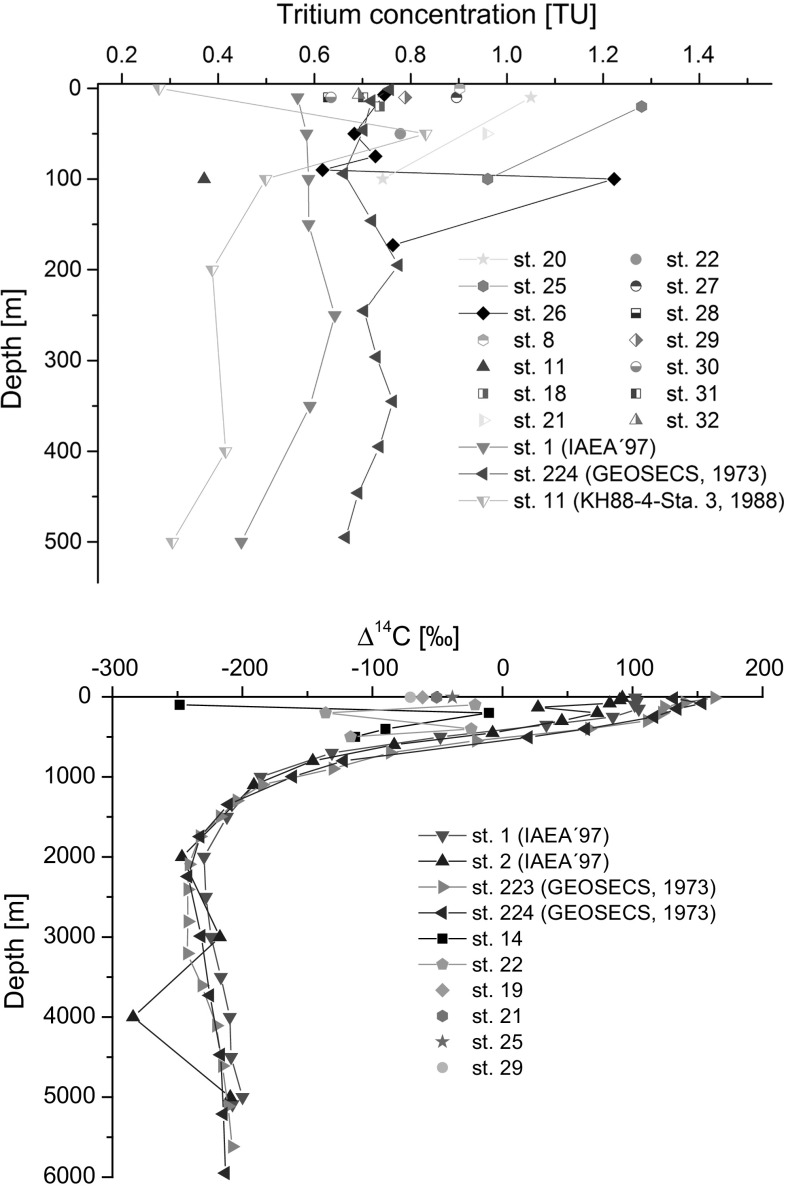
^3^H and ^14^C seawater profiles measured offshore Fukushima NPP during the KoK epedition in June 2011 [103]

From many results, we published recently on Fukushima radionuclides in the Pacific Ocean we present here at least a few data on ^137^Cs, ^3^H and ^14^C in the water column of the NW Pacific. It has been of great interest how Fukushima accident has changed the distribution of these tracers in waters of the open Pacific Ocean, important for climate change studies. The measured ^137^Cs activity concentrations in surface waters collected 30-600 km offshore the Fukushima NPP in June 2011 [4, 102] were up to three orders of magnitude higher than the global fallout background, although the cruise track did not go closer than 30 km from the coast. The geographical distribution of ^137^Cs levels in the water column indicates that an atmospheric deposition of ^137^Cs occurred within the 600 km zone offshore Fukushima. Figure 35 shows as an example the observed ^3^H and ^14^C water profiles with typical higher concentrations at the sub-surface layers. All **Δ**^14^C values observed offshore Fukushima were negative, much lower when compared with our previous investigations [215, 216], as they were influenced (similarly as the ^3^H levels) by Oyashio Intrusion, which brought low radioactivity waters from the north. From the full set of ^3^H/^137^Cs activity ratios observed in the water column, and the previously estimated ^137^Cs releases to the sea [104], we may do the first estimation of the total ^3^H activity released and deposited offshore of the Fukushima coast to be (0.3 ± 0.2) PBq. Measured ^3^H, ^14^C and ^137^Cs concentrations in the NW Pacific Ocean confirm that their distribution has been influenced by the Fukushima accident. Their pre-Fukushima levels, e.g. those previously gathered in the framework of the WOCE and WOMARS projects [217], do not represent anymore the global fallout distribution of these radionuclides in the NW Pacific Ocean.

## Conclusions and outlook

We may summarize that the new developments in ultra-sensitive radioanalytical technologies have had great impact on nuclear and environmental investigations. New applications of radionuclides as tracers of environmental processes were presented in this review, which were not possible before either because of large samples required for radionuclide analyses, or because of limited sensitivities. We illustrated these developments on several topics, however, we could not cover in this review all our previous developments, e.g., on coincidence and multidimensional gamma-spectrometry, on background sources (cosmic rays, cosmogenic, radiogenic and anthropogenic radionuclides) in underground laboratories, on rare nuclear processes, on Monte Carlo simulations, on management of data quality and production of reference materials, etc. Similarly we did not discuss investigations of radionuclides in extra-terrestrial objects (from Luna and Apollo missions to Martian and Lunar meteorites and recently fallen chondrites), cosmic rays and solar variations, radioactive waste dumping sites in the Arctic, Atlantic and Pacific Oceans and adjacent seas, assessment of impacts of nuclear weapons test sites at Mururoa/Fangataufa, Bikini/Enevetak, Algeria and Australia, assessment of environmental impacts from using depleted uranium in warheads (Mediterranean Sea, Adriatic Sea, former Yugoslavia), environmental impacts due to operation of nuclear power plants, global fallout, Chernobyl and Fukushima impacts, submarine groundwater studies, isotope manifestations of climate change studies, to mention at least a few of them. Readers may found references to the corresponding papers in WOS or SCOPUS databases.

We may conclude that the most important development in the radiometric sector has been associated with underground operation of large volume Ge detectors, which have been dominating in analysis of short- and medium-lived radionuclides emitting gamma-rays. This has been mainly because of their high efficiency and excellent resolution, which helped to improve detection limits down to ~ 1 μBq kg^−1^, and to decrease a sample size by about a factor of ten. Specific attention was given to Monte Carlo simulations of background of Ge detectors, which if carried out in advance of the construction of a low-level spectrometry system could predict its parameters. The mass spectrometry sector, represented mainly by AMS and ICPMS revolutionized ultra-sensitive analyses of long-lived radionuclides with detection limits < 1 nBq g^−1^. The ICPMS has proved to be powerful tool because of its high sensitivity, multi-isotopic capability and the low cost per analysis. Nevertheless, there could be problems with molecular, isobaric and isotopic interferences even if careful purification procedures are used. The most exciting breakthrough in the analysis of many long-lived radionuclides has been made in the AMS sector. The AMS systems have been operating at the highest sensitivities with minimum sample size, and small matrix and interference effects. All these new developments in radiometric and mass spectrometry sectors for ultra-sensitive radionuclide analyses have had great impact on investigations of rare nuclear processes and applications in environmental, life and space sciences.

However, there is still a room for new exciting developments which can be documented by the development of new ultra-sensitive laser based analytical techniques, ultra-trace isotope detection of noble gases, development of positive ion sources for tandem accelerators, development of ion traps technologies, to mention at least a few of them. They may include new developments in low energy accelerator/ion trap technologies, or a combination of different analytical technologies including laser based systems. Developments in analytical technologies will further support a transfer from bulk sample analyses to compound specific isotope analyses with on-line coupling of analytical instruments. These new developments would make a single atom counting technology available for many radionuclides, what would be the major achievement in ultra-sensitive analysis of radionuclides, and their wide applications in all sciences based on radionuclide studies. The new technologies will, however, bring new problems, which will require additional attention: (i) A danger of contamination will considerably increase—therefore clean laboratories of class 10–100 will be required; (ii) super-clean chemistry during sample preparation will become even more important; (iii) sample inhomogeneities could mask investigated effects—therefore compound specific isotope analysis of samples will be widely required.

Ultra-sensitive radioanalytical technologies have always been crucial for realization of new scientific ideas in all branches of science. We believe that further developments in new single atom counting technologies will open windows for new even more exciting scientific investigations in the future.
